# Next generation risk assessment: an *ab initio* case study to assess the systemic safety of the cosmetic ingredient, benzyl salicylate, after dermal exposure

**DOI:** 10.3389/fphar.2024.1345992

**Published:** 2024-03-07

**Authors:** Johanna Ebmeyer, Abdulkarim Najjar, Daniela Lange, Mareike Boettcher, Silja Voß, Katrin Brandmair, Jaqueline Meinhardt, Jochen Kuehnl, Nicola J. Hewitt, Christopher-Tilman Krueger, Andreas Schepky

**Affiliations:** ^1^ Beiersdorf AG, Hamburg, Germany; ^2^ Cosmetics Europe, Auderghem, Belgium

**Keywords:** cosmetic ingredients, new approach methodologies, benzyl salicylate, physiologically based pharmacokinetic modeling, *ab initio* safety assessment

## Abstract

We performed an *ab initio* next-generation risk assessment (NGRA) for a fragrance ingredient, benzyl salicylate (BSal), to demonstrate how cosmetic ingredients can be evaluated for systemic toxicity endpoints based on non-animal approaches. New approach methodologies (NAMs) used to predict the internal exposure included skin absorption assays, hepatocyte metabolism, and physiologically based pharmacokinetic (PBPK) modeling, and potential toxicodynamic effects were assessed using pharmacology profiling, ToxProfiler cell stress assay, transcriptomics in HepG2 and MCF-7 cells, ReproTracker developmental and reproductive toxicology (DART) assays, and cytotoxicity assays in human kidney cells. The outcome of the NGRA was compared to that of the traditional risk assessment approach based on animal data. The identification of the toxicologically critical entity was a critical step that directed the workflow and the selection of chemicals for PBPK modeling and testing in bioassays. The traditional risk assessment and NGRA identified salicylic acid (SA) as the “toxdriver.” A deterministic PBPK model for a single-day application of 1.54 g face cream containing 0.5% BSal estimated the C_max_ for BSal (1 nM) to be much lower than that of its major *in vitro* metabolite, SA (93.2 nM). Therefore, SA was tested using toxicodynamics bioassays. The lowest points of departure (PoDs) were obtained from the toxicogenomics assays. The interpretation of these results by two companies and methods were similar (SA only results in significant gene deregulation in HepG2 cells), but PoD differed (213 μM and 10.6 µM). A probabilistic PBPK model for repeated applications of the face cream estimated the highest C_max_ of SA to be 630 nM. The resulting margins of internal exposure (MoIE) using the PoDs were 338 and 16, which were more conservative than those derived from external exposure and *in vivo* PoDs (margin of safety values were 9,705). In conclusion, both traditional and *ab initio* NGRA approaches concluded that the daily application of BSal in a cosmetic leave-on face cream at 0.5% is safe for humans. The processing and interpretation of toxicogenomics data can lead to different PoDs, which can subsequently affect the calculation of the MoIE. This case study supports the use of NAMs in a tiered NGRA *ab initio* approach.

## 1 Introduction

All cosmetic ingredients undergo a safety assessment process to ensure consumer safety. Since the seventh revision (EU, 2003) of the European Directive 76/768/EEC (EU, 2009) was published 20 years ago banning the use of animal studies due to ethical reasons, the cosmetics industry has been a major contributor for the development of new approach methodologies (NAMs). Therefore, next-generation risk assessments (NGRAs) of cosmetic ingredients are based on the strategic use of non-animal methods only, i.e., *in vitro*, *in silico*, and *in chemico* data, in an exposure-led and hypothesis-driven approach to deliver human-relevant safety decisions (Najjar et al., 2023). The acceptance of this requires the demonstration of its consumer-protective application in the form of hypothetical case studies using NGRA principles (Dent et al., 2018). To address this, Cosmetics Europe´s Long Range Science Strategy (LRSS) program included case studies for the NAM-based NGRA to evaluate their practical application for cosmetic ingredients. This approach has been successfully demonstrated in read-across case studies for caffeine (Bury et al., 2021), parabens (Ouedraogo et al., 2022), and phenoxyethanol (OECD, 2021), which have been published in the OECD IATA Toolbox (OECD, 2019a; OECD, 2019b; OECD, 2020). These case studies were based on the SEURAT-1 safety assessment workflow described by Berggren et al. (2017) and Desprez et al. (2018), and according to the International Cooperation on Cosmetics Regulation (ICCR) (Dent et al., 2018).

**FIGURE 1:**
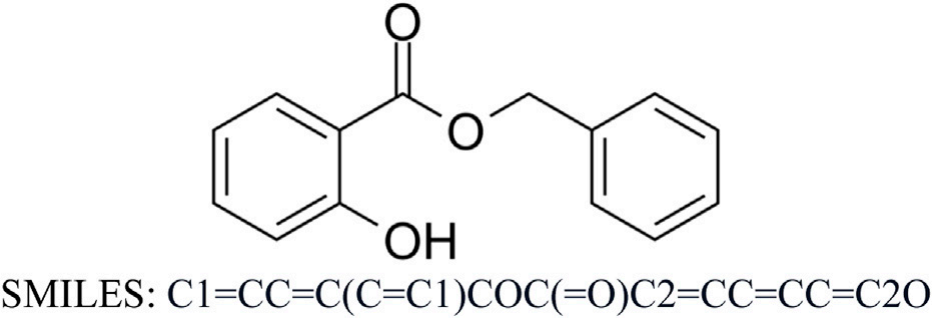
Molecular structure of BSal.

The aim of the current study was to use an NAM-only workflow in an *ab initio* case study as a proof-of-concept to evaluate the readiness of these methods for assessing the systemic safety of the cosmetic ingredients without the use of read-across and exposure-based waiving. Genotoxicity and local effects, e.g., skin irritation and sensitization, were not considered in this assessment since validated NAMs are already available for these endpoints and they were considered by the Scientific Committee for Consumer Safety (SCCS) to not be of concern for the safety assessment for the ingredient evaluated here (SCCS, 2023a; SCCS, 2023c). The cosmetic ingredient used for this case study was benzyl salicylate (BSal), a natural product found in *Desmos chinensis*, *Nicotiana cavicola*, and other organisms (PubChem database). BSal is used in cosmetics as a fragrance ingredient and light stabilizer, with a maximum concentration of 0.5% as a light stabilizer and 0.15% in other leave-on cosmetic products (CIR, 2018). The structure of BSal is shown in Figure 1. This case study set aims to test the current ability to make a safety decision for a cosmetic ingredient without using any *in vivo* data. The safety of this ingredient has been evaluated using traditional risk assessment methods (CIR, 2018; SCCS, 2023a); therefore, *in vivo* legacy information from sources such as the CIR review and SCCS opinion was excluded from the NAM-only *ab initio* workflow and only used to compare the outcome and make a final assessment of the protective value of the NGRA. The selection of a well-established cosmetic ingredient as the subject of the case study enabled the comparison of the NGRA-based safety decision to the traditional (*in vivo*-based) safety assessment outcome.

The overall goal of the case study was to use and evaluate the suitability of NAMs to protect the consumer using a risk-based approach rather than predicting the adverse effects that may be expressed at irrelevant exposure levels to the ingredient. The strategy applied, therefore, involved two main activities. First, collecting or generating a broad suite of bioactivity data in a variety of human-derived ligands and cell lines. Second, employing physiologically based pharmacokinetic (PBPK) modeling to determine whether bioactivity occurs at consumer-relevant concentrations (WHO, 2010). The margins of internal exposure (MoIE) were estimated for BSal using a model to predict blood concentrations following BSal exposure in humans and comparing the results with concentrations that do elicit cellular responses *in vitro*. A similar *ab initio* approach was used by Baltazar et al. (2020) using coumarin as a case study chemical. The MoIE differs from a traditional margin of exposure or margin of safety (MoE/MoS), in that it is calculated as the ratio of a measure of internal exposure, such as blood or target-tissue concentration, rather than comparing external exposure doses (Bessems et al., 2017). The use of a model increases confidence in the risk assessment by incorporating chemical-specific information on the uptake, distribution, metabolism, and excretion of the chemical (Clewell et al., 2008). The overall strategy used was aligned with the United States Environmental Protection Agency’s (US EPA) next-generation blueprint for computational toxicology, which seeks to characterize whether a chemical acts via defined biological pathways/targets or if it may induce cellular changes by a non-specific mechanism (Thomas et al., 2019). Ultimately, the aim of the *ab initio* case study was to support the concept that this approach is “Protection not Prediction” (Thomas et al., 2019), whereby NAMs are used in this case study not to predict a specific toxicity endpoint but to ensure a safe level of exposure which does not result in biologically relevant adverse effects.

## 2 Materials and methods

**FIGURE 2:**
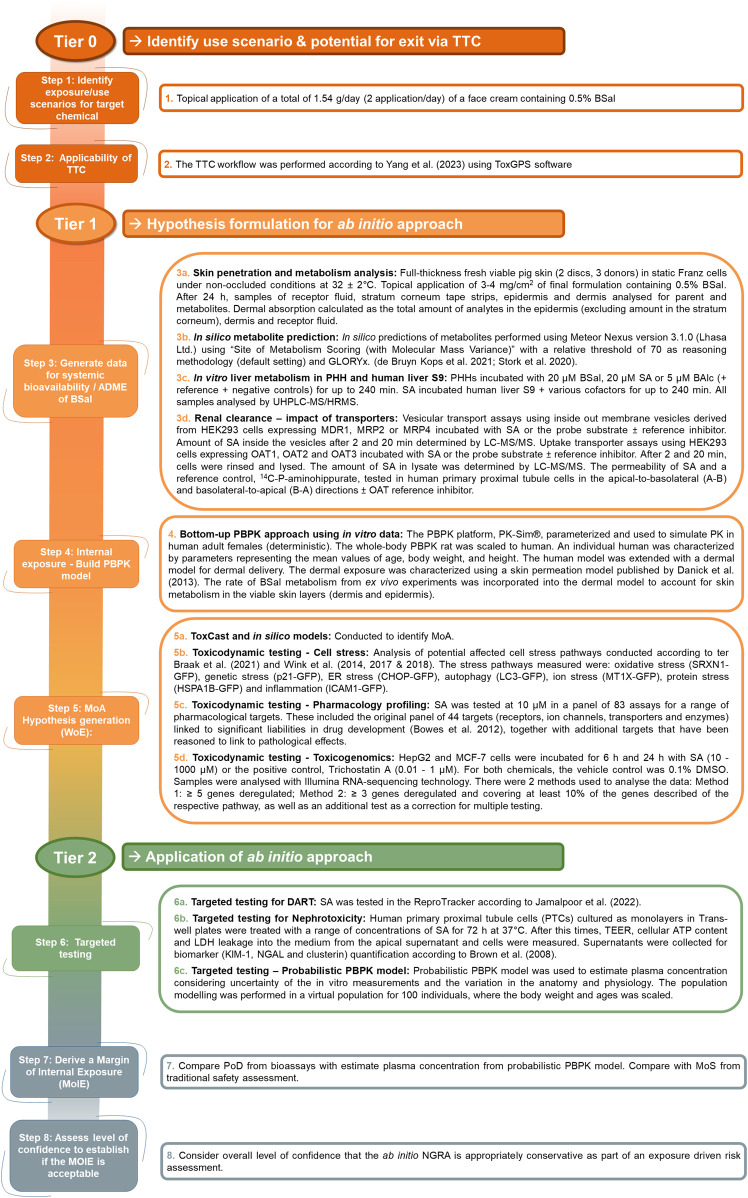
Overview of the *in silico* and *in vitro* methods used in the tiered workflow for the *ab initio* NGRA for BSal.

The safety assessment was conducted in a tiered fashion according to the workflow described by Berggren et al. (2017) and by Desprez et al. (2018). An overview of the *in silico* and *in vitro* methods used in the tiered workflow is shown in Figure 2, and details of the protocols are described in Supplementary Material S2. The key physicochemical properties of BSal and its metabolites that could affect the bioavailability of BSal are listed in Supplementary Table S1.

## 3 Results and discussion of the tiered workflow

### 3.1 Tier 0: identity use scenario, potential for exit via Thresholds of Toxicological Concern

The use scenario selected for the *ab initio* case study was a twice daily topical application of a face cream (a total of 1.54 g/day) containing 0.5% BSal in a human adult population. The calculated relative daily exposure of a face cream is estimated to be 25.6 mg/kg bw/day [based on the application of 1.54 g/day to a 60-kg (default value) adult human (SCCS, 2023c)]. The application of this amount to face skin at 0.5% equates to 128 μg/kg bw/day, which is above the Thresholds of Toxicological Concern (TTC) for non-genotoxic chemicals. The TTC values for systemic toxicity supported by the SCCS for non-genotoxic Cramer Classes I and III compounds are 46 and 2.3 μg/kg bw/day, respectively. The workflow and inclusion criteria for the TTC are described in Supplementary Material S2.2. BSal is not genotoxic, based on *in vitro* assays (Ames test, HPRT test, chromosome aberration *in vitro,* and the *in vitro* micronucleus test) as well as *in silico* predictions (Supplementary Table S2); therefore, the lower TTC of 0.0025 μg/kg bw/day for genotoxic chemicals does not apply. The value of 128 μg/kg bw/day for BSal is, however, greater than the TTC value for systemic toxicity for non-genotoxic Cramer Classes III (2.3 μg/kg bw/day). In conclusion, the calculation of the daily exposure indicated that the TTC could not be applied, and more investigations are required.

### 3.2 Tier 1: hypothesis formulation for an *ab initio* approach

#### 3.2.1 Systemic bioavailability: skin penetration and metabolism

In studies using fresh pig skin (see Supplementary Material S2.3 for the method), 24 h after the application of a formulation containing 0.5% BSal, there were several analytes identified in the epidermis, dermis, and receptor fluid. These included the parent compound, BSal, as well as salicylic acid (SA), benzoic acid (BA), benzyl alcohol (BAlc), and hippuric acid (Table 1), as well as traces of Phase II conjugates of BSal. After 24 h, the majority of the BSal was recovered during the skin wash as the unchanged parent compound and very little of it penetrated the epidermis or dermis as the parent compound (dermal penetration was 2.2% as BSal only). Of note, only two analytes reached the receptor fluid, namely, SA and BAlc, with the levels of the parent chemical below the LOQ. This indicates that the extensive first-pass metabolism of BSal occurs in the skin. BSal was present in the epidermis and dermis; therefore, both BSal and its metabolites can be assumed to enter the systemic circulation after topical application.

**TABLE 1:** Absorption and metabolism of BSal 24 h after the application of 0.5% BSal in formulation to the viable pig skin.

Fresh porcine skin	Benzyl salicylate	Salicylic acid	Benzoic acid	Benzyl alcohol	Hippuric acid	Benzaldehyde
Surface	63.8 ± 2.4	0.3 ± 1.2	1.7 ± 1.2	< LOQ*	< LOQ*	< LOQ*
Stratum corneum	1.2 ± 0.3	0.3 ± 0.1	16.8 ± 16.3	< LOQ*	0.15 ± 0.17	< LOQ*
Epidermis	1.2 ± 0.1	0.2 ± 0.1	< LOQ*	0.1 ± 0.0	0.01 ± 0.01	< LOQ*
Dermis	1.0 ± 0.4	3.4 ± 0.4	1.2 ± 0.7	1.8 ± 0.3	0.05 ± 0.07	< LOQ*
Receptor fluid	< LOQ*	3.3 ± 0.4	< LOQ*	1.7 ± 0.1	< LOQ*	< LOQ*
Total recovery	67.2 ± 2.6	7.5**	19.8 ± 17.2	3.6 ± 0.5	0.2 ± 0.15	< LOQ*
Dermal absorption of individual analytes	2.2 ± 0.2	6.9 ± 0.4	1.2 ± 0.7	3.6 ± 0.5	0.26 ± 0.07	< LOQ*

Values are expressed as a percentage of the applied dose and are a mean ± SD, n = 6 skin discs (three donors). * LOQ: limit of quantification, ** total recovery was corrected by the amount of SA present in the formulation itself.

The total dermal penetration of BSal and SA was 9.1% of the applied dose. The mass balance was 74.7% when BSal and SA were included and increased to 90.8% with integration of additional metabolites of BSal. This result is in line with the OECD 428 test guideline, “Skin Absorption: *in vitro* Method.” Additionally, the measured dermal penetration and recovery data for BSal are in accordance with data derived from studies on frozen human skin using radioactive BSal [9.01% of the applied dose (SCCS, 2023a)].

#### 3.2.2 Systemic metabolism and elimination: *in silico* metabolite prediction

**FIGURE 3:**
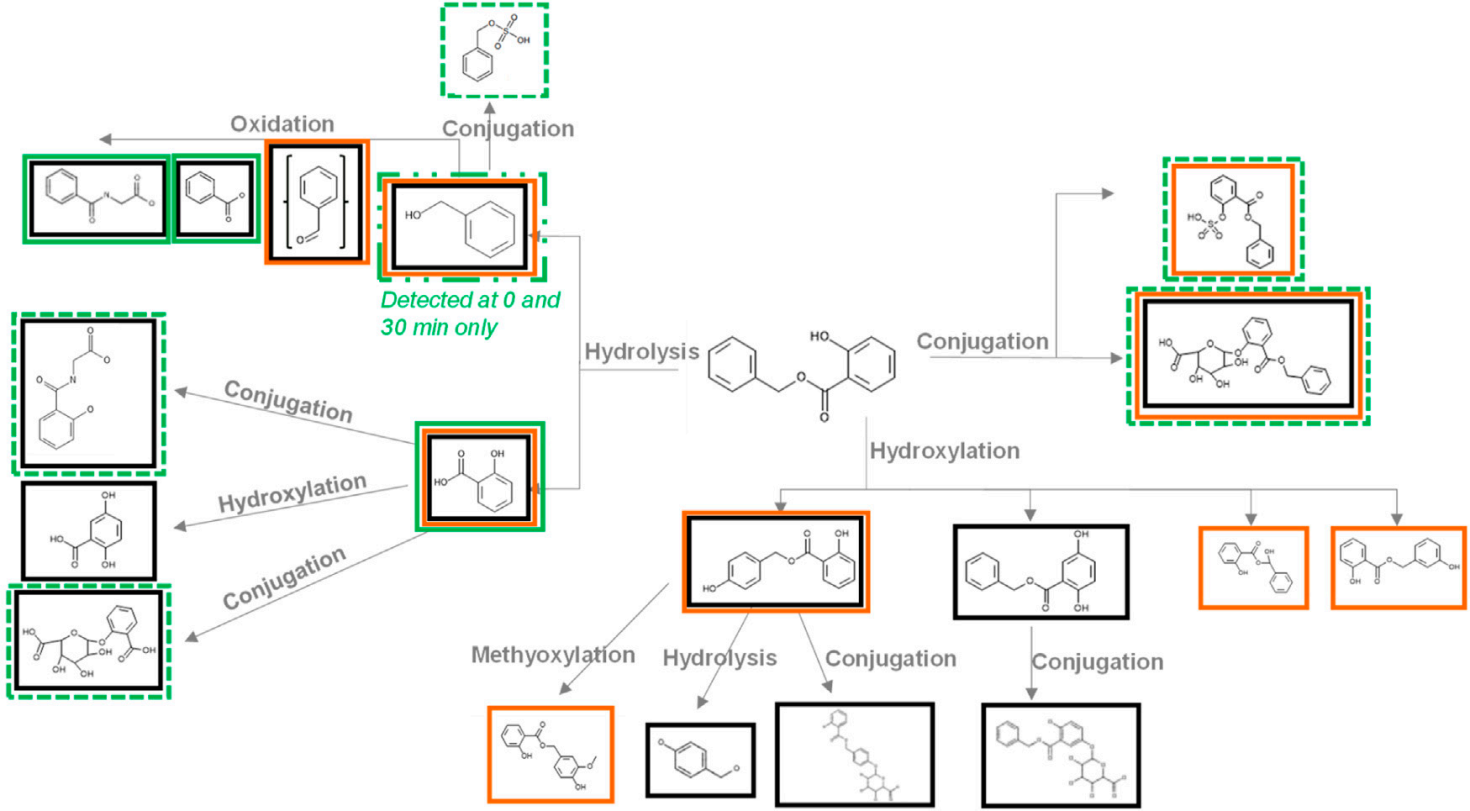
Metabolites predicted by Meteor and GLORYx and detected during the incubations of BSal with PHH. Metabolites in black boxes were predicted by Meteor, metabolites in orange boxes were predicted by GLORYx, and metabolites in green boxes were detected in incubations with PHH (a dashed green line denotes metabolites identified by accurate mass only and are probably only present in trace amounts).

Meteor and GLORYx (Supplementary Material S2.4) were used to predict the potential metabolites formed from BSal (Figure 3). The software predicted multiple metabolites via different pathways, which included the following: (1) hydrolysis of BSal to SA and BAlc, with further metabolism of BAlc to benzaldehyde, BA and, finally, hippuric acid, plus further Phase II conjugation or hydroxylation of SA; (2) Phase II conjugation of BSal; and (3) hydroxylation of BSal. The latter was assessed to be of minor relevance since hydrolysis (i.e., pathway A) takes place very rapidly and thus is much more likely. The Extended Clearance Classification System (ECCS) was implemented to predict the predominant clearance mechanism based on the physicochemical properties and passive membrane permeability of the parent and the metabolites (Varma et al., 2015). The ECCS predicts BSal and BAlc as class 2 [metabolism as the primary clearance mechanism (high-permeability neutrals)] and SA and BA as class 1A [metabolism as the primary systemic clearance mechanism (high-permeability acids with molecular weight (MW) ≤400 Da).

#### 3.2.3 Systemic metabolism and elimination: *in vitro* liver metabolism

**FIGURE 4:**
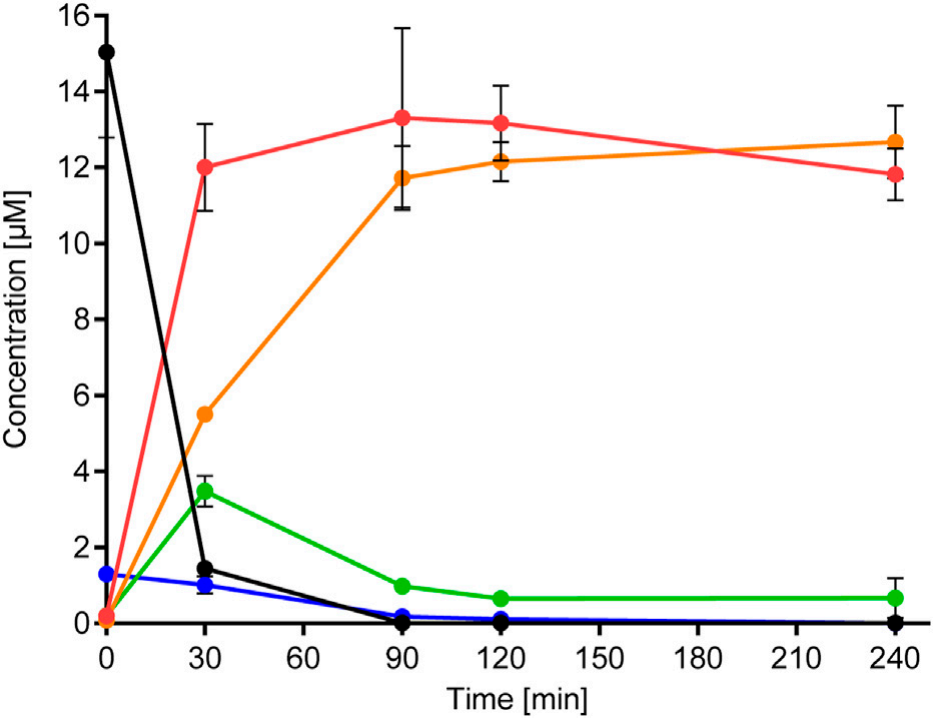
Metabolism of BSal and formation of metabolites in PHH. Values are indicated as mean ± SD of triplicate samples. Black symbols/lines denote BSal, red symbols/lines denote SA, orange symbols/lines denote hippuric acid, green symbols/lines denote BA, and blue symbols/lines denote BAlc.

The metabolism of BSal by primary human hepatocytes (PHHs) was very rapid, with only 10% of the initial concentration remaining after 30 min (Figure 4, see Supplementary Material S2.5 for the method). Despite the rapid metabolism, 0.1%–0.2% of the initial concentration was detected in the PHH after 90, 120, and 240 min. The half-life of BSal in the PHH was 9.2 min, with an *in vitro* intrinsic clearance (CL_int,_ _*in vitro*_) of 94.2 μL/min/million cells. Based on the *in vitro* results, the estimated half-life of SA was >950 min (∼16 h), with a CL_int,_ _*in vitro*_ of ∼0.9 μL/min/million cells.

Figure 3 shows the results of the prediction models Meteor and GLORYx and compares these with those of the metabolites detected in hepatic incubations with BSal. *In vitro* results correlated with the predicted hydrolysis pathway, with the exception that SA metabolites were not observed *in vitro*. The main pathway in the PHH was hydrolysis to SA and benzylic metabolites (BA, BAlc, and hippuric acid, shown in Figure 4), as well as trace amounts of Phase II conjugates. SA was the main metabolite, the formation of which mirrored the depletion of the parent compound (Figure 4). BAlc was detected in the first timepoint, following which it decreased and was not detected after 240 min. This is in accordance with the results from additional incubations of BAlc with the PHH, which indicate its rapid metabolism within 30 min. BA was formed from BSal, with a peak concentration at 30 min, but BA was quickly further metabolized to hippuric acid (the final detoxified metabolite which is expected to be excreted). Notably, SA was not metabolized further under these conditions, which was also confirmed in separate incubations of SA with the PHH and liver S9 assay (see Supplementary Material S2.6 for the liver S9 method and Supplementary Material S2.7 for the analytical methods and calculations).

The conclusions from the *in vitro* hepatic incubations are that (a) BSal that reaches the systemic circulation is rapidly metabolized to SA and benzylic metabolites; (b) benzylic metabolites do not reach high concentrations and are rapidly further metabolized to hippuric acid, which is regarded as an excretion metabolite; (c) SA is stable under these conditions and is, thus, a relevant metabolite; (d) there was a good comparison between the predicted metabolites using *in silico* models (Figure 3) and the major metabolites formed in *in vitro* incubations with the PHH (Figure 4), providing confidence that relevant metabolites were accounted for in the safety assessment.

#### 3.2.4 Systemic elimination: renal clearance

Since SA is a stable main metabolite, its excretion was investigated using different uptake and efflux transporter studies (see Supplementary Material S2.8 for the method). These assays revealed that SA was not a substrate of the human ABC (efflux) transporters: MDR1, MRP2, and MRP4 or the human SLC (uptake) transporters: OAT1 and OAT2v1. SA may be a borderline substrate of the tOAT3 SLC transporter since, although the fold accumulation was above the 2-fold cut-off at 10 µM and 2 min, the fold accumulation was below the 2-fold cut-off at the rest of the test conditions (data not shown).

Incubations with fresh primary human proximal tubule cells (hPTCs) indicated that there was an increase in the movement of SA across the monolayers over time with increasing concentrations (1–100 µM), suggesting transcellular transport (Supplementary Figure S1A). However, SA did not appear to move predominantly in either direction or preferentially accumulate in the cells, and the concentration had no impact on the direction of the flux (Supplementary Figure S1D). Coincubation with probenecid, an inhibitor of OATs and MRP2, tended to increase permeation in the absorptive direction (A–B). This effect was marginal but statistically significant and consistent at 10 and 100 µM and may in part be due to inhibition of OATs at the basolateral membrane of the hPTCs. This effect was transient at the lower concentration but was sustained at a higher concentration, with no net movement at 10 µM after 120 min with probenecid and sustained absorptive movement at 100 μM, suggesting that this is a dose-dependent and transporter “drug–drug interaction.” The intracellular concentration was not influenced by inhibition of the transporters.

#### 3.2.5 Internal exposure: PBPK modeling

**FIGURE 5:**
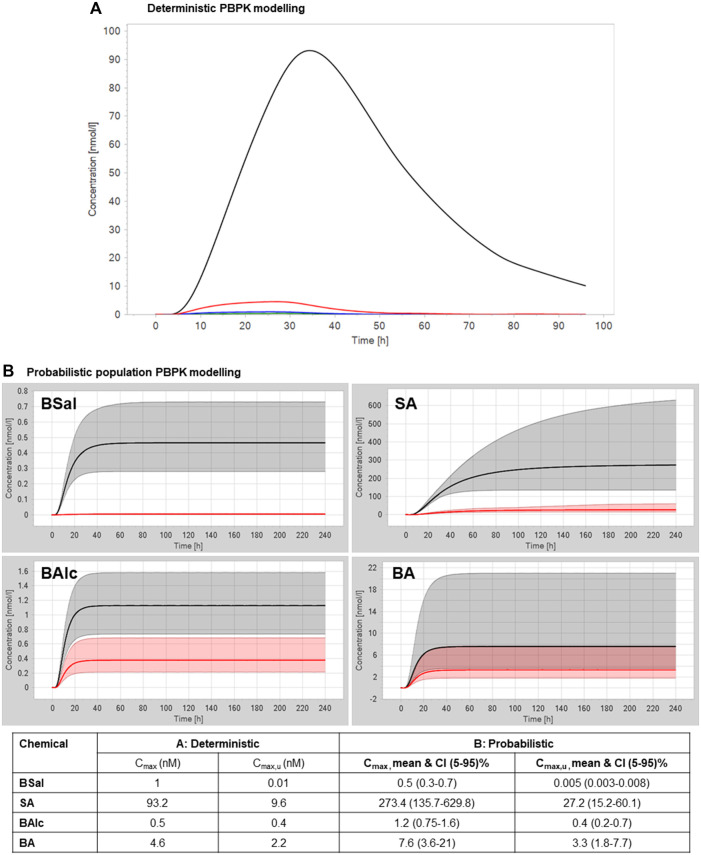
**(A)** Deterministic PBPK modeling for plasma concentrations of BSal and its metabolites after a single-day application of 1.54 g face cream containing 0.5% BSal. The green line represents BSal, the black line represents SA, the blue line represents BAlc, and the red line represents BA. **(B)** Probabilistic PBPK modeling for plasma concentrations of BSal and its metabolites after repeated application of 1.54 g/day face cream containing 0.5% BSal. The black line represents the total geometric mean, the red line represents the unbound geometric mean, the gray shaded areas represent the 5%–95% total plasma concentration range, and the red shaded areas represent the 5%–95% unbound plasma concentration range.

In a deterministic PBPK model for the human adult and the physicochemical properties listed in Supplementary Table S1, an approximate estimation of the plasma concentrations of the most relevant parent/metabolites after a single-day application of 1.54 g/day face cream containing 0.5% BSal to an adult human was made. This resulted in total C_max_ values of 1 nM for BSal, 93.2 nM for SA, 0.5 nM for BAlc, and 4.6 nM for BA (Figure 5A). These values were used to conclude that (a) BSal should not be tested further since the exposure to this was negligible compared to that of SA and (b) that SA should be tested in bioassays and the concentration of 93.2 nM expected *in vivo* should be included in the testing to ensure that relevant concentrations were used. The total C_max_ for SA was ∼90-fold higher than that of BSal; in addition, the fraction unbound in the plasma (Fup) of BSal and SA is 0.66% and 2.9%–18%, respectively. This results in higher exposure of unbound SA compared to unbound BSal by at least ∼5-fold. Therefore, SA was considered to be the most relevant metabolite of BSal from a systemic exposure point of view. Hence, SA was selected for testing in toxicodynamics bioassays.

#### 3.2.6 Interim conclusion on exposure assessment

BSal is metabolized in the skin to SA, BAlc, BA, and hippuric acid. However, some BSal reaches the systemic circulation. Systemically bioavailable BSal is rapidly metabolized in the liver to SA and benzylic metabolites. Benzylic metabolites do not reach high concentrations and are rapidly further metabolized to hippuric acid, which is regarded as an excretion metabolite. The deterministic PBPK model resulted in SA concentrations approx. 90 times higher than those of BSal. Thus, SA is considered the most relevant metabolite for safety assessment. Therefore, toxicodynamics assays were performed with SA.

#### 3.2.7 MoA hypothesis generation (WoE): ToxCast and *in silico* models

BSal and its metabolites detected in incubations with the PHH were evaluated using *in silico* and *in vitro* tools to identify potential toxic effects and the mode of action (MoA). *In silico* alerts [using Derek Nexus, QSAR toolbox, Case Ultra, and ADMET predictor (see Supplementary Material S2.9 for information on the model versions)] and in *in vitro* test results for BSal and its metabolites, as well as estrogen-, androgen-, and thyroid signaling and steroidogenesis (EATS) assay results from the ToxCast database, are summarized in Supplementary Tables S2 and S3, respectively. Both *in silico* alerts and *in vitro* tests indicated that BSal was non-genotoxic but that it has the potential to interact with the endocrine system via the estrogen pathway [it was positive in several *in vitro* ToxCast assays for the estrogen receptor (ER), androgen receptor (AR), and steroidogenesis but not for the thyroid pathway]. However, BSal was positive in 9 out of 18 ER assays and was positive in 1 out of 14 AR assays, where the lowest reported AC_50_ was 36.5 µM in a binding assay for ERα (assay name: OT_ER_ERaERa_0480). Based on *in silico* alerts only, BSal and its metabolite, SA, were indicated to cause reproductive and developmental toxicity. SA, however, was indicated to be non-genotoxic and not an endocrine disruptor based on *in silico* alerts and *in vitro* tests. Additional *in silico* alerts indicated that SA causes nephrotoxicity and hepatotoxicity. The potential for nephrotoxicity was followed up in a targeted test (see Section 3.3.2, targeted testing: nephrotoxicity). Hippuric acid was negative in all 259 ToxCast assays. The potential for hepatoxicity was addressed by the cell stress panel, which uses HepG2 cells, a liver tissue-derived cancer cell line (Section 3.2.8) and by toxicogenomics testing in HepG2 cells (Section 3.2.10). The developmental and reproductive toxicology (DART) alert was followed up by performing the ReproTracker assay (Section 3.3.1).

SA was considered to be the most important molecule to investigate based on the *in silico* alerts for toxicity and on its predominant exposure levels compared to BSal and its other metabolites.

#### 3.2.8 Toxicodynamic testing: cell stress

Saturation of cellular stress pathways can result in a host of adverse health effects including embryotoxicity, neurotoxicity, and hepatotoxicity (Simmons et al., 2009). SA was tested over a concentration range of 10 μM to 10 mM in the ToxProfiler cell stress assay (Supplementary Material S2.10). The ToxProfiler assay is based on a panel of seven HepG2 reporter cell lines, analyzing chemically induced activation of seven distinct stress pathways by GFP-tagged biomarkers of these pathways. The following pathways were studied using the respective biomarkers: oxidative stress (SRXN1-GFP), genetic stress (p21-GFP), ER stress (CHOP-GFP), autophagy (LC3-GFP), ion stress (MT1X-GFP), protein stress (HSPA1B-GFP), and inflammation (ICAM1-GFP).

SA was not cytotoxic up to the highest tested concentration and activated only ICAM1, a stress pathway reporter linked to inflammation (Supplementary Figure S2). The observed response, although significant, was marginal (a ratio of <0.1 when normalized to GFP responses) compared to the responses to the assay’s reference compounds. Based on these observations, the PoD for SA was derived to be 4,000 μM; however, there was only a slight increase followed by a decrease in the signal at the next higher incubation dosage; therefore, the effect may not be biologically relevant. Furthermore, these results indicate that the *in silico* alert for hepatotoxicity (Section 3.2.7) was not supported by *in vitro* data.

#### 3.2.9 Toxicodynamic testing: pharmacology profiling

SA was subjected to a panel of 83 target ligand-binding assays (see Supplementary Material S2.11 for details). The panel based on the safety pharmacological profiling panel as published by Bowes et al., used 44 targets associated with *in vivo* adverse drug reactions pertaining to the central nervous system, cardiovascular, pulmonary, and gastrointestinal toxicities (Bowes et al., 2012). The set of targets was extended to broaden the biological coverage. The experiments used an SA concentration of 10 µM and encompassed two technical replicates. Results showing an inhibition or stimulation higher than 50% were considered to represent the effects of the test compound, and responses between 25% and 50% indicated some but less certain effects, both results requiring follow-up testing in this NGRA-based, *ab initio* case study. Acetylcholinesterase is the most sensitive target, albeit the inhibition was only 25.4%. In a follow-up study, concentrations of 10 μM to 1 mM SA inhibited acetylcholinesterase by even less (only up to 5%). A literature search indicated that SA is a very weak COX inhibitor (Patrignani and Patrono, 2015); therefore, despite not being a hit in the screening assay (10 µM did not inhibit COX1 or COX2), concentrations up to 10 mM were tested for their ability to inhibit COX1 and COX2. This assay showed that SA did not inhibit COX1 up to 10 mM and inhibited COX2 by 29% at 10 mM. It was not possible to calculate an IC_50_ for COX2 (which was set at > 10 mM).

#### 3.2.10 Toxicodynamic testing: toxicogenomics

**FIGURE 6:**
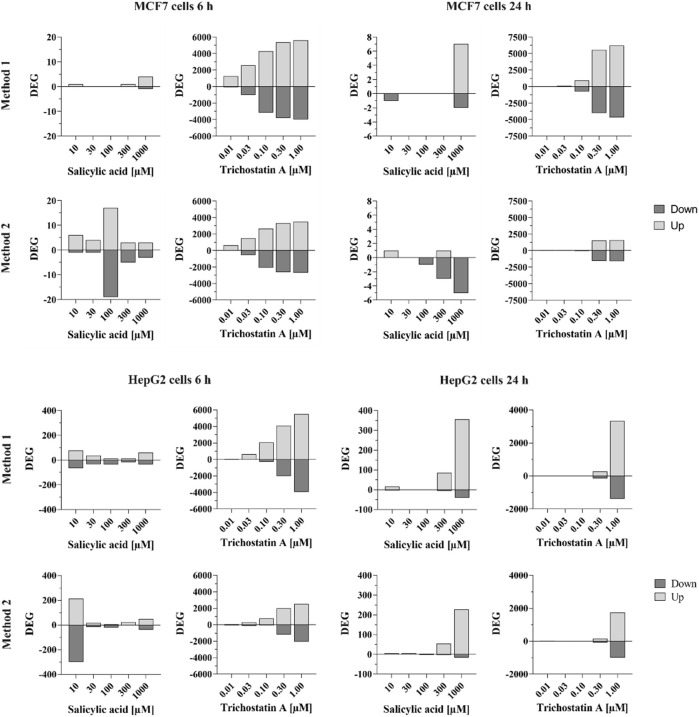
Number of significantly differentially expressed genes in MCF-7 and HepG2 cells treated with SA or trichostatin A for 6 or 24 h according to analysis method 1 (top) or analysis method 2 (bottom). Differentially expressed genes (up- or down-regulated) are shown with absolute fold-change ≥ ±1.5 and Benjamini–Hochberg-corrected *p*-value (false discovery rate, FDR) FDR <0.05 (analysis method 1) or Global Permutation-based *p*-value ≤0.005 (analysis method 2).

HepG2 and MCF-7 cells were treated with SA up to 1 mM with 0.1% DMSO used as a vehicle control. As observed in the cell stress assay using HepG2 reporter cell lines (Section 3.2.8), SA was not cytotoxic up to the highest tested concentration in the HepG2 and MCF-7 cells used in this assay. Whole-genome transcriptomics dose response analysis was performed to derive a pathway-based no-observed-transcriptional-effect level (NOTEL, referred to as PoD) (according to (Lobenhofer et al., 2004)) for SA in HepG2 and MCF-7 cells (see Supplementary Material S2.12 for details). The raw data were evaluated by two analysis methods with slightly different statistical approaches. In both cases, 1.5-fold deregulation was used as a cutoff for identifying the number of up- and downregulated differentially expressed genes (DEGs). Analysis Method 1 considered Benjamini–Hochberg-corrected *p*-values as the false discovery rate (FDR) with a cutoff of FDR <0.05. Analysis Method 2 identified 1.5-fold deregulated genes with a global permutation-based *p*-value ≤0.005. For more details of the analysis methods, see Supplementary Material S2.12. Figure 6 shows the comparison of the number of statistically significant DEGs according to the above criteria in HepG2 and MCF-7 cells after 6- and 24-h treatment and analyzed using both methods. Cells were treated with SA or the positive control, trichostatin A. Trichostatin A is an antibiotic antifungal known to impact gene expression by inhibiting mammalian histone deacetylases (Vanhaecke et al., 2004). Thus, strong gene expression changes were expected.

Trichostatin A resulted in clear concentration-dependent increases in the number of DEGs at both timepoints in both cell lines. The responses were similar using both analysis methods, although more genes were affected, with a gradual increase across the concentrations, when the data were analyzed with Method 1.

Analysis Method 1 resulted in very few genes in MCF-7 cells that were statistically significantly altered by SA. For example, only five and nine genes were altered by > 1.5-fold or < -1.5-fold in MCF-7 cells after treatment with 1,000 µM SA after 6 and 24 h, respectively, compared to ∼10,000 altered by trichostatin A at 1 μM at both timepoints (analysis Method 1). There was no concentration-dependent effect of SA on MCF-7 cells observed using analysis Method 1; therefore, data from this cell line were excluded from the derivation of the PoD.

**FIGURE 7:**
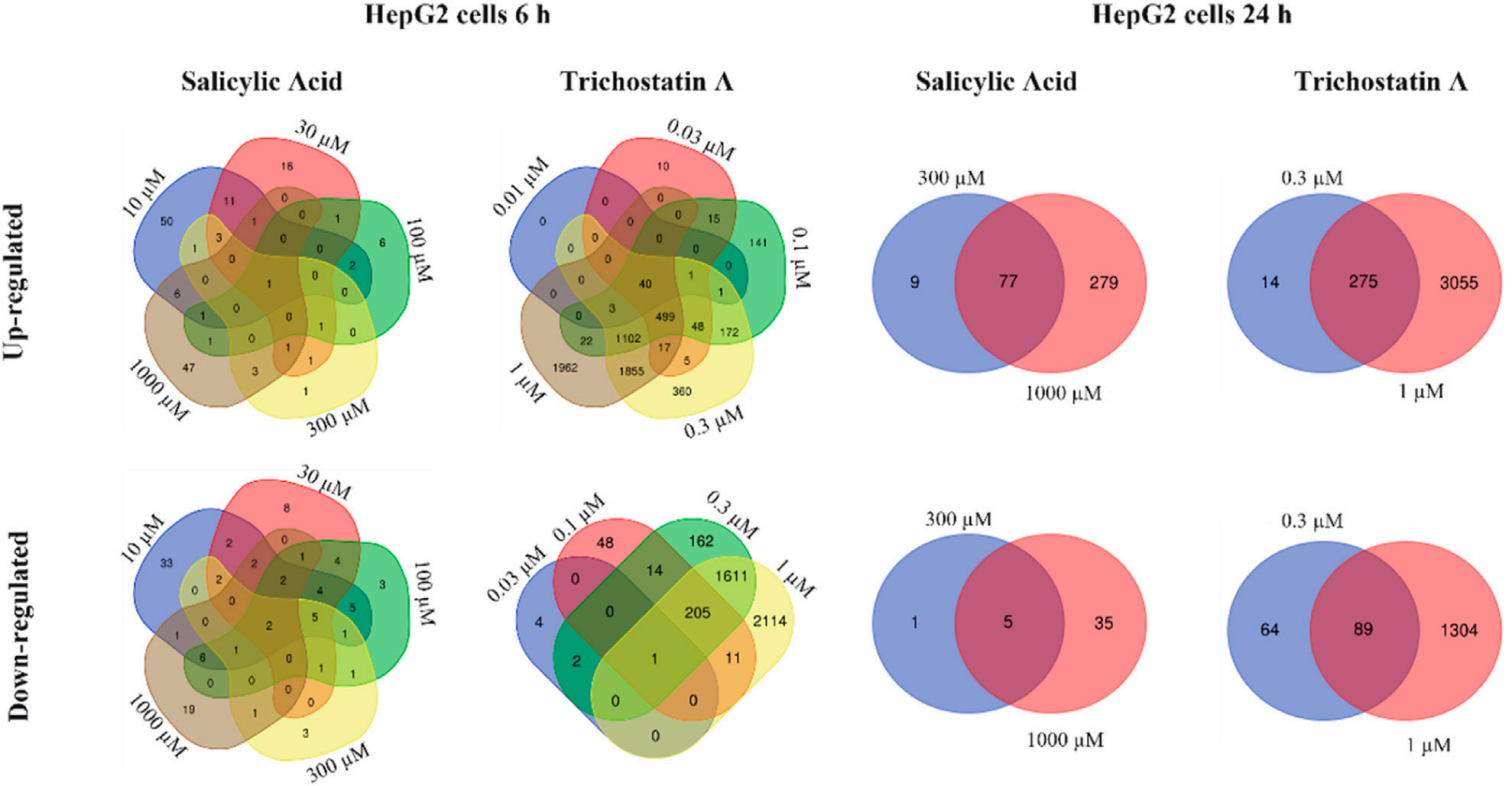
Venn diagrams showing the overlap of similar up- and down-regulated genes for different concentrations of SA and trichostatin A in HepG2 cells incubated for 6 and 24 h.

Relatively more genes were statistically significantly altered by SA in HepG2 cells, the number of which was higher after 24 h. After 6 h, the number of DEGs increased with increasing concentrations, apart from the 10 µM concentration. However, almost none of the identified significantly DEGs were deregulated by more than one concentration (each concentration affected different individual genes) as shown in Venn diagrams analyzing the overlap of similar deregulated genes at different concentrations (analysis Method 1, Figure 7). In contrast to the 6 h timepoint, at 24 h, almost all the genes that were differentially deregulated by 300 µM SA were also differentially deregulated by 1,000 µM (analysis Method 1, comparing Venn diagrams analyzing the overlap of similar deregulated genes at the different concentrations in Figure 7). This indicated that the response using HepG2 cells incubated with SA for 24 h was concentration-dependent and could be used to derive the PoD. The positive control trichostatin A, which is expected to cause a concentration-dependent effect, clearly showed similar DEGs.

Analysis using Method 2 showed similar trends compared to analyses using Method 1, but with different absolute numbers. Again, only very few DEGs were observed in MCF-7 cells, both after 6-h and 24-h treatment. In HepG2 cells, there were more DEGs observed. As with the analysis using Method 1, there was no concentration-dependent effect observed after 6-h treatment but after 24-h treatment. Thus, benchmark dose analysis was performed for both analysis methods based on the HepG2 24-h dataset.

In order to derive a potential PoD, benchmark dose modeling was performed. BMD modeling was performed with HepG2 incubated for the 24-h dataset since only this dataset showed a concentration-dependent effect of SA using analysis Method 1 (see above). In both, analyses Method 1 and Method 2, base line +1 SD was considered as a benchmark response. For considering a conservative approach, the PoD was derived from the lowest pathway affected using the lower 95% confidence limit of the benchmark dose. In analysis Method 1, any ontology category using Gene Ontology terms and Reactome with at least five or more best model elements found among the defined ontology category elements and with Fisher’s exact test *p*-value <0.05 was considered enriched. The genes associated with each enriched pathway were then used to estimate the pathway or category BMD_1SD_ and BMDL. Analysis Method 2 considered GO terms and Reactome pathways with the following criteria: Fisher’s exact right tail *p*-value ≤0.05, pathway coverage ≥10%, and the number of significant dose responsive genes in pathway ≥3. Base line +1 SD was considered to be the BMD. Based on these criteria, PoDs of 213 µM (analysis Method 1) and 10.6 µM (analysis Method 2) were derived. The marked difference in the two analysis methods is most likely based on the different inclusion criteria (five genes vs. three genes +10% coverage).

### 3.3 Tier 2: application of an *ab initio* approach

#### 3.3.1 Targeted testing: ReproTracker

The ReproTracker assay was performed due to the DART alert for SA (see Supplementary Material S2.13 for the method). ReproTracker is a human-induced pluripotent stem cell (hiPSC)-based biomarker assay that assesses various key events during early embryonic development. A potential impairment of key developmental stages is analyzed by morphological changes and the expression of specific marker genes in hiPSCs differentiated into cardiomyocytes, hepatocytes, or neural rosettes. SA had no significant effect on the expression pattern of the biomarker genes in all three cell types up to the concentrations of 1 mM (Supplementary Figure S3). SA was, therefore, classified as not teratogenic up to 1 mM. The highest dose tested (1 mM) resulted in morphological changes in hepatocytes and neural rosettes, as well as a slight, but not significant decrease of one cardiomyocyte marker (BMP4). Based on these effects, a PoD of 500 µM was set for this endpoint.

#### 3.3.2 Targeted testing: nephrotoxicity

The nephrotoxicity of SA was evaluated due to the nephrotoxicity alert from *in silico* predictions (using Derek). A comprehensive evaluation of the nephrotoxicity was conducted using hPTCs cultured in a transwell format (see Supplementary Material S2.14 for the method). SA was not toxic with respect to TEER, LDH release, or ATP content at any of the concentrations tested up to 300 μM (data not shown). Clinical biomarkers were also used to determine the nephrotoxicity, none of which were markedly or concentration-dependently affected (data not shown).

#### 3.3.3 Targeted testing: probabilistic PBPK model

To account for the uncertainty of the *in vitro* measurements and the variation in anatomy and physiology, a probabilistic PBPK model was used to estimate the plasma concentration considering in a virtual population of 100 individuals (Najjar et al., manuscript in preparation). The virtual population was built based on a range of body weights and ages. The uncertainty of the *in vitro* parameters was with respect to the standard deviations to define the range of these parameters rather than using the mean values. The relevant PK parameters were reported, considering the mean and confidence intervals 5%–95% that were used later for the MoIE calculations. The resulting population PBPK modeling (together with the values from deterministic modeling) of plasma concentrations of BSal and its metabolites after a repeated application of 1.54 g/day face cream containing 0.5% BSal is shown in Figure 5B. The higher spread of data is due to the large difference in plasma protein-binding values (which ranged from 3 to ∼20% for SA), which has a big impact on the renal filtration of SA (as the only route of elimination). The highest mean plasma concentration was for SA (273.4 nM), which was over 500-fold higher than the predicted mean C_max_ for BSal. This value of 273.4 nM SA was used in the calculation of the MoIE. The model already has uncertainties built into it; therefore, this value includes several assumptions that were discussed (data not shown, manuscript is in preparation).

#### 3.3.4 PoDs, IVIVE, uncertainty estimation, and MoIE

**FIGURE 8:**
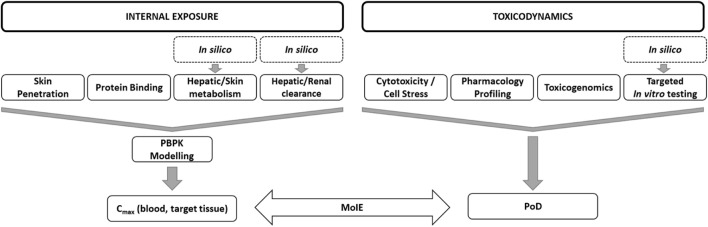
Overview of the tiered workflow and the related *in silico* and *in vitro* NAMs used in the NGRA.

Figure 8 provides an overview of the tiered workflow and the related *in silico* and *in vitro* NAMs used to calculate the MoIE. Several MoIE values were derived from PoDs from the *in vitro* toxicodynamics assays and plasma concentrations predicted using PBPK modeling (Table 2 and Table 3). Since this assessment is conducted using plasma concentrations rather than external doses, the comparison is an MoIE. We selected the two lowest PoDs from the *in vitro* bioassays, which were based on the toxicogenomics results evaluated by Analysis Method 1 (213 µM) and Analysis Method 2 (10.6 µM) since it is not yet known as to which of the two methods is more appropriate.

**TABLE 2:** Summary of PoDs for SA derived from *in vitro* bioassays and used in the NGRA.

Assay	PoD	Based on endpoint
Cell stress—ToxProfiler cell stress assay	4,000 µM	ICAM1 (indicating inflammation)
Pharmacology profiling	≥10,000 µM	COX1/2
Toxicogenomics	213 μM (method 1)	Concentration-dependent gene deregulation in HepG2 cells at 24 h
	10.6 µM (method 2)	
ReproTracker	500 µM	Morphological changes, BMP4
Nephrotoxicity	>300 µM	Highest dose tested

**TABLE 3:** Calculation of MoIE for the NGRA.

Lowest PoD	PoD (µM)	PBPK model	C_max_ [mean, µM]	MoIE	C_max_ [CI95, µM]	MoIE
BMDL analysis method 1	213	Probabilistic	0.273	780	0.630	338
BMDL analysis method 2	10.6	Probabilistic	0.273	38	0.630	16

In this assessment, the total plasma concentrations were used, rather than unbound concentrations, because they are compared with nominal doses used in the *in vitro* assays (biokinetics in the toxicogenomics incubations were not measured). When the mean total C_max_ concentration of 0.273 µM was compared to the PoDs, the MoIEs were 780 and 38 for PoDs of 213 and 10.6 µM, respectively. When the more conservative CI95 value of 0.630 µM was used for the calculation of the MoIE, the values decreased further, with the lowest value of 16 derived using the Analysis Method 2 PoD of 10.6 µM.

#### 3.3.5 Traditional risk assessment

For comparison with the MoIE values derived using an NGRA, MoS values were also calculated using the traditional approach with legacy *in vivo* data to determine the PoD and a calculation of the exposure according to the external dose and dermal absorption data (Table 4). The explanation of the selection of the values used in the calculation is detailed in the SCCS opinion on BSal (SCCS, 2023a). For BSal present at 0.5% in a face cream, the SCCS based the PoD on SA since BSal is rapidly metabolized to this primary stable metabolite. Despite the results from *in vitro* metabolism studies and PBPK population modeling indicating that BSal does enter the systemic circulation, the maximum concentration (CI95) of 0.7 nM BSal was much lower than that for SA (630 nM), indicating that SA is the driver of systemic effects. Therefore, the lowest PoD for SA was taken from a reproductive toxicity study on rats in which an NOAEL of 75 mg/kg bw was derived based on teratogenic and embryotoxic effects (SCCS, 2023b). Correction factors for the molecular weight (converting SA and other metabolites to BSal), skin first-pass metabolism (assuming 100% conversion), and the route-to-route exposure (which is 1 since the route is oral for BSal and SA) were applied to the PoD. The systemic exposure dose (SED) was calculated by adjusting the amount of the product applied with the amount absorbed after topical application. As shown in Table 3, the MoS value for BSal was 9,705.

1) The NOAELs for the safety comparator were obtained from the literature: SA from SCCS (2023b); 2) adjustment of metabolites to the parent by accounting for the molecular weight; 3) complete metabolism of BSal to SA; 4) dermal absorption: metabolite plus BSal plus + 1SD each; 5) rounded to 123 mg/kg/day by SCCS.

**TABLE 4:** Calculation of the MoS using the traditional approach using *in vivo* PoDs and external exposure.

	Parameter	Value
PoD	Comparator chemical	Salicylic acid
	NOAEL of safety comparator^1^ [mg/kg/day]	75
	Correction factor^2^ (MW)	228.25/138.12 = 1.65
	Correction factor [route-to-route]^3^	1
	Corrected NOAEL [mg/kg/day]	75 × 1.65 = 123^5^
Exposure	Amount of product^3^ [mg/kg/day]	24.14
	Concentration [%]	0.5
	Dermal absorption^4^ [%]	10.5
	SED total [mg/kg/d]	24.14 × 0.5/100 × 10.5/100 = 0.01267
MoS		123.8/0.01267 = 9,705

## 4 Discussion

Since the European ban on the use of animal testing to evaluate the safety of cosmetic ingredients, there have been significant advances in the development and strategic use of NAMs to achieve the same goal. While validated NAMs are available to evaluate the hazard potential of cosmetic ingredients to cause local effects, e.g., skin and eye irritation and skin sensitization, the potential of chemicals to cause systemic and long-term toxicity requires a more complex strategy, i.e., an NGRA (SCCS, 2023c). This takes into account human-relevant effects and relates them to exposure to derive a safe concentration of a cosmetic product component which is protective to human health. We followed the tiered NGRA testing strategy described by Berggren et al. (2017) and Desprez et al. (2018) and recommended in the SCCS Notes of Guidance. While a read-across strategy was possible for BSal, we investigated the level of protection to human health when an *ab initio* assessment was conducted because future cosmetic ingredients may not have the luxury of structural or biological analogs with which to conduct a read-across assessment. The levels of protection according to the NGRA approach (i.e., MoIE) using NAMs and using a traditional MoS calculation using animal data were evaluated. The results of the tiered workflow are summarized in Table 5.

**TABLE 5:** Summary of data used in the case study.

Data	Result
Tier 0: Identity use scenario, potential for exit via TTC	
Identification of use scenario	Topical application of 1.54 g/day face cream containing 0.5% BSal
Case study type	*ab initio* approach, use of *in vivo* data only for comparison traditional risk assessment vs *ab initio* approach
TTC	TTC not applicable since external exposure is 128 µg/kg bw/day and higher than the thresholds of 46 and 2.3 µg/kg bw/day for non-genotoxic Cramer Classes I and III compounds, respectively.
Tier 1: Hypothesis formulation for *ab initio* approach	
Systemic bioavailability: Skin penetration and metabolism analysis	In studies with fresh porcine skin, of 0.5% BSal in an emulsion (body lotion) resulted in detection of the analytes salicylic acid (SA), benzyl alcohol (BAlc), benzoic acid (BA) and hippuric acid identified in the epidermis, dermis and receptor fluid, as well as traces of phase II conjugates. Dermal penetration was: 2.2% as BSal; 9.1% as BSal + metabolites. The mass balance was 90.8% as BSal + metabolites. These findings were in line with data in human skin studies (SCCS, 2023a).
Systemic metabolism & elimination*: In silico* metabolite prediction	Several metabolites of BSal predicted:
	A: hydrolysis of BSal to SA and BAlc. Further metabolism of BAlc to benzaldehyde, BA and, finally, hippuric acid + further phase II conjugation or hydroxylation of SA
	B: Hydroxylation of BSal assessed to be of minor relevance since hydrolysis takes place very rapidly and thus is much more likely.
	C: Phase II conjugation of BSal
	*In vitro* results fit very well to metabolism pathway A (with the exception that SA metabolites are not observed *in vitro*). The Clearance Classification System (ECCS) predicts BSal and BAlc as class 2 (metabolism as primary clearance mechanism (high permeability neutrals), whereas SA and BA as class 1A (metabolism as primary systemic clearance mechanism (high permeability acids with molecular weight (MW) ≤400 Da).
Systemic metabolism & elimination: *In vitro* liver metabolism	BSal is rapidly metabolized in primary human hepatocytes (PHH). The main pathway is hydrolysis into SA and benzylic metabolites, as well as trace amounts of phase II conjugates. SA is not further metabolized (also confirmed in separate incubations of SA only in PHH and liver S9); however, BA is formed and then quickly further metabolized into hippuric acid (the final detoxified metabolite which is excreted). Additional incubations of BAlc with PHH indicate it is very rapidly metabolized within 30 min.
	The half-life of BSal was 9.2 min, with an *in vitro* intrinsic clearance of 94.2 µl/min/million cells. The half-life of SA was calculated 16.4 h, with an *in vitro* intrinsic clearance of 0.9 µl/min/million cells.
Systemic elimination: Renal clearance	SA was not a substrate of the human ABC (efflux) transporters: MDR1, MRP2 and MRP4 or the human SLC (uptake) transporters: OAT1 and OAT2v1. SA may be a borderline substrate of OAT3 SLC transporter. Therefore, only glomerular filtration rate (GFR) was considered for the renal clearance of SA.
Internal exposure: PBPK modelling	A deterministic PBPK model for a single application of 1.54 g/day face cream containing 0.5% BSal exposure scenario was generated. This PBPK model applied only default values to determine an approximate plasma concentration estimate for the most relevant parent/metabolites. The modelling resulted in C_max_ values of 1 nM for BSal, 93.2 nM for SA, 0.5 nM for BAlc and 4.6 nM for BA. Due to the high factor of 90, only SA was considered relevant for testing in toxicodynamics bioassays (although concentrations for both chemicals were predicted to be below the SCCS interim iTTC value of 1 µM (SCCS, 2023c)).
Interim conclusion on exposure assessment	BSal is metabolized in the skin to SA, BAlc, BA and hippuric Acid. However, some BSal reaches the systemic circulation. Systemically bioavailable BSal is fast metabolized in the liver to SA and benzylic metabolites. Benzylic metabolites do not reach high concentrations and are rapidly further metabolized to hippuric acid, which is regarded as an excretion metabolite. The deterministic PBPK model resulted in SA concentrations approx. 90 times higher compared to BSal. Thus, SA is considered as the most relevant metabolite for safety assessment. Therefore, toxicodynamics assays were performed with SA.
*MoA Hypothesis generation (WoE):* ToxCast and *in silico* models	SA: alerts for developmental toxicity, reproductive toxicity, nephrotoxicity, hepatotoxicity, Hippuric acid: negative in all 259 ToxCast assays.
*Toxicodynamic testing:* Cell stress	SA, was tested in a concentration range of 10 µM to 10 mM in the ToxProfiler cell stress assay. A POD of 4000 µM based on ICAM1 (indicating inflammation) was identified, however, there was only slight increase followed by a decrease in the signal; therefore, the effect may not be biologically relevant.
*Toxicodynamic testing:* Pharmacology profiling	SA tested at 10 µM in a panel of 83 assays for a range of pharmacological targets. 10 µM SA inhibited acetylcholinesterase by 25.4 %, which was the most sensitive target affected. Follow-up dose-response curves showed no effect on COX1 up to 10 mM and 29% inhibition of COX2 at 10 mM. 10 µM to 1 mM SA inhibited acetylcholinesterase by ≤5%.
*Toxicodynamic testing:* Toxicogenomics	Whole genome transcriptomics dose response analysis performed for SA in HepG2 and MCF-7 cells and raw data evaluated by 2 different methods. Very few genes in MCF-7 cells were statistically significantly altered by SA. Relatively more genes were statistically significantly altered by SA in HepG2 cells. A concentration-dependent effect of SA was observed at the 24 h timepoint only, whereas most altered genes at 6 h indicated a non-specific transcriptome response. The response using HepG2 cells at 24 h was used to derive PoDs based on lower confidence limit of the benchmark dose (BMDL) of 213 μM (analysis method 1) and 10.6 µM (analysis method 2). The difference between the two evaluations was most likely due to different criteria considering a pathway as relevant for BMD analysis (method 1: ≥ 5 genes deregulated; method 2: ≥ 3 genes deregulated and covering at least 10% of the genes described of the respective pathway).
Tier 2: Application of *ab initio* approach	
*Targeted testing*: ReproTracker	The ReproTracker assay was performed due to the *in silico* DART alert of SA (which is in line with the CMR classification of SA but pretended to be unknown for the ab initio risk assessment approach). According to the ReproTracker results, SA was classified as not teratogenic up to 1 mM. Morphological changes were observed at the highest dose tested (1 mM), as well as a slight decrease of one cardiomyocyte marker (BMP4). A PoD of 500 µM was set based on the morphological changes at the higher concentration.
*Targeted testing*: Nephrotoxicity	Nephrotoxicity of 1 – 300 µM SA was investigated using human proximal tubular cells (hPTCs). SA was not toxic with respect to TEER, LDH release or ATP content at any concentration. Clinical biomarkers of nephrotoxicity were also not markedly or concentration-dependently affected.
*Targeted testing*: Probabilistic PBPK model	Probabilistic PBPK model was used to estimate the highest mean plasma concentration of SA to be 273.4 nM.
*PODs, IVIVE, Uncertainty estimation, MOS*	Lowest PoDs were from the toxicogenomics assays: 213 µM and 10.6 µM. MoIEs were 338 and 16 using probabilistic simulations, CI95 C_max_. These margins of safety were more conservative than those derived from external exposure and *in vivo* PoDs (MoS values were 9705).

A critical aspect of the safety assessment was the identification of the toxicologically relevant entity since this dictates which chemical(s) (parent and/or metabolites) should be tested in the toxicodynamics bioassays. Tier 1 skin absorption and metabolism experiments using *ex vivo* pig skin indicated that there was extensive first-pass metabolism in the skin after application of the intended dose of BSal in a formulation and that almost all of BSal was metabolized to several metabolites. The dermal bioavailability was determined (*in vitro*) to be 9.1% by summing up the amount of BSal and salicylic acid reaching the living skin layers and receptor fluid. These results using fresh pig skin correlated well with the results from a study using frozen human skin (and ^14^C-radio-labelled BSal present in a formulation at 0.5%), in which the skin absorption was 9.01% of the applied dose (SCCS, 2023a). Pig skin is accepted as a suitable surrogate for human skin (OECD, 2004; SCCS, 2023c) and has been shown by others to generate comparable skin absorption and metabolism results with those in human skin (Gerstel et al., 2016; Géniès et al., 2019). This was the first evidence in the current case study that subsequent *in vitro* bioassays should not focus on BSal, but rather its metabolites.

Metabolite prediction models help confirm results obtained in *in vitro* metabolism studies, or they can provide an indication of potential metabolites before the assays are run (which helps with searching for specific masses in the MS analysis). In this study, Meteor and GLORYx software were used to predict BSal metabolites. Both models predicted three possible pathways (hydrolysis, conjugation, and hydroxylation), with one or more metabolites representing these pathways flagged by both models. Considering the high capacity of the skin and liver for hydrolyzing esters (Jewell et al., 2007), it was concluded that this would be the main pathway involved in BSal metabolism. Indeed, there was a good concordance between the results obtained using Meteor and GLORYx and those from *in vitro* incubations of BSal with PHH, including the direct conjugation of BSal, as well as its hydrolysis to SA and benzyl derivatives. One exception was that further metabolites of SA predicted by Meteor and GLORYx were not observed in the *in vitro* incubations with the PHH, possibly due to the short duration of the incubation (4 h). Therefore, for PBPK modeling, it was assumed that SA is excreted via glomerular filtration despite the knowledge that, in humans, SA is partly excreted as conjugates (glucuronide and glycine conjugates) and as an oxidized metabolite (gentisic acid) (Needs and Brooks, 1985). Since this is an *ab initio* study, the literature information was not used in the PBPK model. In terms of the safety assessment, the assumption that SA is not metabolized is a conservative strategy since further metabolism of SA would decrease systemic concentrations and thus increase the MoIE. Future studies could investigate the use of long-term incubation models for hepatic metabolism, e.g., sandwich cultures and liver spheroids, if no metabolism of test chemicals is observed after 4-h incubation with PHH suspensions.

A second major metabolite formed in incubations of BSal with PHH was BA. The formation of this metabolite peaked at 30 min but was quickly further metabolized to hippuric acid, which continued to be formed until all the BSal had been consumed (by ∼2 h). BAlc and virtually all benzyl derivatives are readily oxidized to BA, which is then conjugated with glycine to produce hippuric acid, a detoxified metabolite (Irwin et al., 2016). In theory, according to the *ab initio* concept, the detoxification of BA occurring *in vivo* would not be known; therefore, it was necessary to predict the plasma concentrations of BA as well as SA in the next stage of the case study.

Since NGRAs are exposure-driven, PBPK modeling now represents a key aspect of the quantitative exposure assessment to investigate the kinetics of parent chemical and metabolite ADME parameters. PBPK modeling allows ADME parameters to be followed over time, in contrast to traditional risk assessments, in which only static parameters are used (exposure is calculated by considering the NOEAL and % absorbed). Therefore, PBPK modeling provides a more precise overview of the fate of the parent test chemical and its metabolites, leading to a more reliable safety assessment. In the first step, the PBPK model incorporated mean values in a deterministic approach. This supported the conclusion that BSal would be present in the systemic circulation at very low concentrations (1 nM). The hydrolysis product, BAlc, and its oxidized metabolite, BA, were also predicted to be present at low concentrations (0.5 and 4.6 nM, respectively). In contrast, SA was predicted to be present in plasma at a much higher concentration of 93.2 nM. Based on this simulation, only SA was tested in the *in vitro* bioassays. Of note, a concentration of 93.2 nM is much lower than the interim iTTC of 1 µM recommended by the SCCS (SCCS, 2023c) and, therefore, could have been used to conclude that neither the parent test chemical nor its major metabolite, SA, exceeded the iTTC, indicating there was no toxicological concern of BSal under the use condition being evaluated. However, the ToxCast and *in silico* toxicity prediction models indicated that SA has potential for developmental and reproductive toxicity, which excludes this from the iTTC approach.

Probabilistic PBPK modeling was considered to estimate the range of C_max_ by considering the probable distribution of the input parameters, which reflect the uncertainty in the *in vitro* measurements and variation in human physiology and anatomy. This resulted in similar plasma concentrations for BSal, BAlc, and BA, but higher values for SA: a mean of 273.4 nM. PBPK models can also be refined with respect to the contribution of uptake and efflux transporters to excretion, and since SA was identified as being metabolically stable, additional studies were conducted to determine whether it is a substrate for transporters known to be expressed in the kidney. Transporter studies using hPTCs suggested that transport was mainly transcellular. This meant that renal clearance was not adjusted according to transporters but was based on the glomerular filtration rate and F_up_. This is considered to be protective of human health because lack of transporter-mediated uptake into the kidney results in lower excretion of SA and a higher concentration in plasma. Of note, uncertainty factors for PBPK modeling were already incorporated in the model, e.g., the high range of values for F_up_ for the analytes and the assumption that SA is excreted unchanged in the urine.

In the Tier 1 hypothesis generation, *in silico* alerts and *in vitro* tests indicated that BSal has potential endocrine disruption effects via the estrogen pathway. By contrast, the *in silico* alerts and *in vitro* tests indicated that the major metabolite and comparator chemical, SA, is not an endocrine disruptor. The lowest AC_50_ for BSal was 36.5 µM in a binding assay for ERα, which is > 52,000-fold higher than the CI95 C_max_ for BSal. Therefore, it was concluded that BSal would not cause endocrine disruption at the concentrations predicted in the plasma after the application of BSal in a face cream.

In the initial stage of Tier 1 hypothesis generation, ToxCast data and the *in silico* models indicated that SA was linked to nephrotoxicity and hepatotoxicity. Therefore, targeted testing explored whether SA was toxic to primary human kidney cells (hPTCs) according to standard viability parameters and clinically relevant biomarkers. This was also addressed in Tier 1, using the ToxProfiler cell stress assay, which was conducted as a general marker of systemic toxicity, rather than a targeted test in Tier 2 targeted testing [but did provide an indication of the potential for both nephrotoxicity and hepatotoxicity (Simmons et al., 2009)]. These assays ruled out the liver and kidney as target organs for SA effects, with PoDs in the millimolar range. Even if hepatotoxic effects are caused by SA, they are covered within the assessment by the assays conducted (the ToxProfiler assay indicated only a very slight effect in HepG2 cells) and the final PoD, which was based on toxicogenomics in HepG2 cells.

Pharmacology profiling and toxicogenomics assay were run to investigate a potential MoA for SA. The targets used in the pharmacology profiling assay panel were designed to provide a broad biological space (Burbank et al., 2022). The original panel comprised 44 targets and was designed for pharmaceuticals. This panel was extended to include additional targets, including kinases, as well as targets related to DART (ERα/β, PPARα/γ/δ), hepatotoxicity (e.g., BSEP), and cardiotoxicity (e.g., ion channels). This approach that helps identify targets has been advocated by the SCCS in the most recent Notes of Guidance, with the aim of increasing confidence in an NGRA (SCCS, 2023c). In the current study, none of the targets tested were significantly impacted by SA at a concentration up to millimolar concentrations.

The only assay in which PoDs were in the micromolar range was from non-targeted testing toxicogenomics assays using HepG2 cells. In contrast, the effects of SA on MCF-7 cells were minimal (very few genes deregulated) and not concentration-dependent and were thus attributed to non-specific transcriptome changes. An interesting aspect of this case study was the interpretation of the same set of toxicogenomics raw data by two biostatisticians from two companies. This is important because the way in which data are processed and interpreted can result in differences in the overall conclusion of an NGRA, as was demonstrated in this study whereby the MoIE differed by 20-fold, depending on the PoD used. The PoD derived from HepG2 cells incubated with SA for 24 h was 213 µM according to Analysis Method 1 and 10.6 µM according to Analysis Method 2. The difference between the two evaluations was due to the different number of genes per pathway used (Method 1: ≥5 genes deregulated; Method 2: ≥3 genes deregulated and covering at least 10% of the genes described of the respective pathway, as well as an additional test as a correction for multiple testing). Neither approach is incorrect; they simply differ in the criteria used to derive the PoD. This highlights the need to standardize data processing or at least provide transparent processes to be able to track how the data were handled. Regarding the transparent tracking of processes used, the US EPA has developed a so-called generic “Transcriptomics Reporting Framework” (TRF) to address this issue (Gant et al., 2017). The TRF describes raw data selection, data normalization, recognition of outliers, and statistical analysis.

This case study originated from the idea of comparing a traditional safety assessment with an NGRA assessment. Within this investigation, transcriptional changes have proven to be the most sensitive parameter and were used as the PoD, with the caveat that the interpretation of these transcriptional changes with respect to their links to toxicity remains uncertain. However, this approach was adopted to comply with the concept of protectiveness. When the CI95 was used to predict the internal exposure, the MoIE was 338 calculated using the PoD of 213 µM from BMDL Method 1 and 16 using the PoD of 10.6 µM from BMDL Method 2. There is no current agreement on the required minimum MoIE in NGRAs to assess a compound as safe and, while some have reported MoIEs greater than 100 using a similar approach to that in the current case study (Baltazar et al., 2020), this might not be necessary, considering the conservativeness of NAM-based approaches. Others have suggested the use of a so-called “Bioactivity/Exposure Ratio” (BER), which is equivalent to the MoIE and indicates whether the use of an ingredient is safe or not—the higher the BER, the less likely the systemic concentration will be bioactive and potentially cause adverse effects (SCCS, 2023c). The use of the BER is being increasingly used by others, e.g., the US EPA (Harrill, 2018), Health Canada (Health Canada, 2021), and ICCR principles (Dent et al., 2018; Carmichael et al., 2022). Regardless of which nomenclature is used, it is still to be determined what value constitutes a suitable threshold above which a chemical can be considered to be unlikely to cause adverse effects to human health. It is hoped that case studies like this will help refine and/or add weight to the values already implemented by others.

Since the PoD used here was based on a change in gene expression rather than an apical adverse effect, it could be assumed that the *ab initio* NGRA for BSal is sufficiently conservative to assure consumer safety. In addition, PBPK modeling is evolving to such an extent that there is more confidence in the predictions, which means that the use of the upper CI95 concentration as an estimation of the exposure may be an over-conservative prediction of the internal concentration or that a minimum MoIE needed to reach a safety decision should be re-defined. This is in accordance with a recent report in which high-throughput safety assessments based on *in vitro* PoDs covering a broad biological space and exposure predictions were sufficiently protective in most cases and were at least as protective as using *in vivo* PoDs (Paul Friedman et al., 2020). When a traditional approach used in the BSal opinion of the SCCS (SCCS, 2023a) using *in vivo*-derived PoDs and exposure calculated from the external dose and percentage absorbed, the MoS for the same consumer use (i.e., application of 0.5% BSal in a face cream) was 9,705. This indicates that while both assessments identified SA as the toxdriver, the *ab initio NGRA* approach, based on the comparison of internal exposure and *in vitro*-derived PoDs, was more conservative compared with the traditional method to ensure consumer safety.

## 5 Conclusions on BSal based on this case study

In conclusion, we have demonstrated that both traditional and *ab initio* NGRA approaches concluded that the use of BSal in a cosmetic leave-on face cream at 0.5% is safe for humans. While we used default safety assessment factors for the traditional approach, we purposefully chose higher levels of conservatism in applying the NGRA approach. This pertains especially to parameters chosen for the PBPK model as the outcome from this model is critical to estimate the internal exposure concentrations for the MoIE calculations. On the other hand, the identification of a suitable margin to depict human safety requires additional insights. For this case study, the identification of the toxicologically critical entity was a critical step that directed the workflow and the selection of chemicals that were considered in PBPK modeling and tested in bioassays. Both the traditional risk assessment and NGRA identified SA as the “toxdriver.”

The overall conclusions from toxicogenomics assays by two companies were similar in terms of identifying that SA only results in significant gene deregulation in HepG2 cells at 24 h. Importantly, the raw data processing and interpretations highlighted how these can lead to different PoDs, which can subsequently affect the calculation of the MoIE. Thus, there is an urgent need to define clear rules and approaches for PoD derivation from toxicogenomics data.

This case study based on BSal supports the use of NAMs in a tiered NGRA *ab initio* approach.

## Figures and Tables

**Table:** 

**ADME**	Absorption, distribution, metabolism, and excretion
**AR**	Androgen receptor
**BMD**	Benchmark dose
**BMDL**	Lower 95% confidence limit of the benchmark dose
**BSal**	Benzyl salicylate
**BA**	Benzoic acid
**BAlc**	Benzyl alcohol
**C**_**max**_	Maximum plasma concentration
**DART**	Developmental And Reproductive Toxicology
**DEGs**	Differentially expressed genes
**EATS**	Estrogen-, androgen-, thyroid signaling, and steroidogenesis
**ER**	Estrogen receptor
**FDR**	False discovery rate
**Fup**	Fraction unbound in the plasma
**ICCR**	International Cooperation on Cosmetics Regulation
**LOEL**	Lowest observed effect concentration
**LogP**	Logarithm of octanol/water partition coefficient
**MCF7**	Breast cancer cell line
**MoA**	Mode of action
**MoE**	Margin of exposure
**MoIE**	Margin of internal exposure
**MoS**	Margin of safety
**NAM**	New approach methodology
**NOAEL**	No observed adverse effect level
**NOTEL**	No-observed-transcriptional-effect level
**PBPK**	Physiologically based pharmacokinetic modeling
**PHH**	Primary human hepatocytes
**PoD**	Point of departure
**PPAR**	Peroxisome proliferator-activated receptor
**SA**	Salicylic acid
**SCCS**	Scientific Committee for Consumer Safety
**TTC**	Threshold of Toxicological Concern
**US EPA**	United States Environmental Protection Agency

## Data Availability

The original contributions presented in the study are included in the article/Supplementary Material; further inquiries can be directed to the corresponding author.
