# Supplementary material for: Next generation risk assessment: an ab initio case study to assess the systemic safety of the cosmetic ingredient, benzyl salicylate, after dermal exposure
Source: Front Pharmacol. 2024 Mar 7;15:1345992. doi: 10.3389/fphar.2024.1345992 (PMC10955127; doi:10.3389/fphar.2024.1345992)
Supplement: Supplementary file 3 [file DataSheet1.docx]

Supplementary material

**S1 Supplementary Tables and Figures**

**S1.2 Supplementary Tables**

**Supplementary Table S1. Physicochemical properties of BSal and its metabolites relevant to bioavailability.** Values were from PubChem or the ECHA database

| **Property** | **Benzyl salicylate** | **Benzyl alcohol** | **Benzoic acid** | **Salicylic acid** |
| --- | --- | --- | --- | --- |
| CAS number | 118-58-1 | 100-51-6 | 65-85-0 | 69-72-7 |
| MW | 228.24 | 108.14 | 122.12 | 138.12 |
| Water solubility (mg/ml) | 8.8 | 40 | 3.5 | 138.12 |
| Partition coefficient (Log Pow) at 35°C | 4 | 1.05 | 1.88 | 2.64 |
| pKa at 25°C | 9.82 | 15.4 | 4.19 | 2.8 |
| Melting point (°C) | < -50 | -15.4 | 122 | 157 to 160 |
| Boiling point (°C) | 322 | 205.3 | 249.2 | 256 |
| Vapor pressure (Pa) | 0.01 | 7 | 0.1 | 0.0208 |
| Density (g/ml) | 1.181 | 1.045 | 1.321 | 1.44 |

**Supplementary Table S2.** *In silico* Toxicity tools (ToxPanel) of case study compounds (Benzyl salicylate + relevant metabolites Salicylic acid, Benzyl alcohol, Benzoic acid) – EXCEL CHART

**Supplementary Table S3.** EATS assay results from the ToxCast database – EXCEL CHART

**1.2 Supplementary Figures**

**Supplementary Figure S1. Flux of SA across hPTCs at 1 µM (A), 10 µM (B) and 100 µM (C) in the absence and presence of probenecid and intracellular accumulation (D).** In A-C, light grey bars are absorptive flux (J_a-b_), dark grey bars are secretory flux (J_b-a_), and the black bars represent the net flux. Data are expressed as the mean (± S.E.M.) of three technical replicates from three donors. Significance is denoted by: * = P < 0.05, ** = P < 0.01, *** = P < 0.001, **** = P < 0.0001.

**Supplementary Figure S3.** Analysis of morphological changes and expression of specific marker genes in hiPSCs differentiated into cardiomyocytes, hepatocytes and neural rosettes treated with the test compound, benzyl salicylate. All experiments were performed in two biological replicates in the presence of the test compounds. Positive (Thalidomide) and negative (Saccharin) controls were included in every experiment.

**S2 Supplementary Materials and Methods**

**S2.1 Chemical sourcing**

Benzyl salicylate, salicylic acid, benzaldehyde, benzoic acid and hippuric acid were all purchased from Sigma Aldrich. Benzyl alcohol was from Acros Organics.

**S2.2 Workflow and inclusion criteria for the TTC**

The TTC workflow was performed according to Yang et al. (2023) using ToxGPS software.


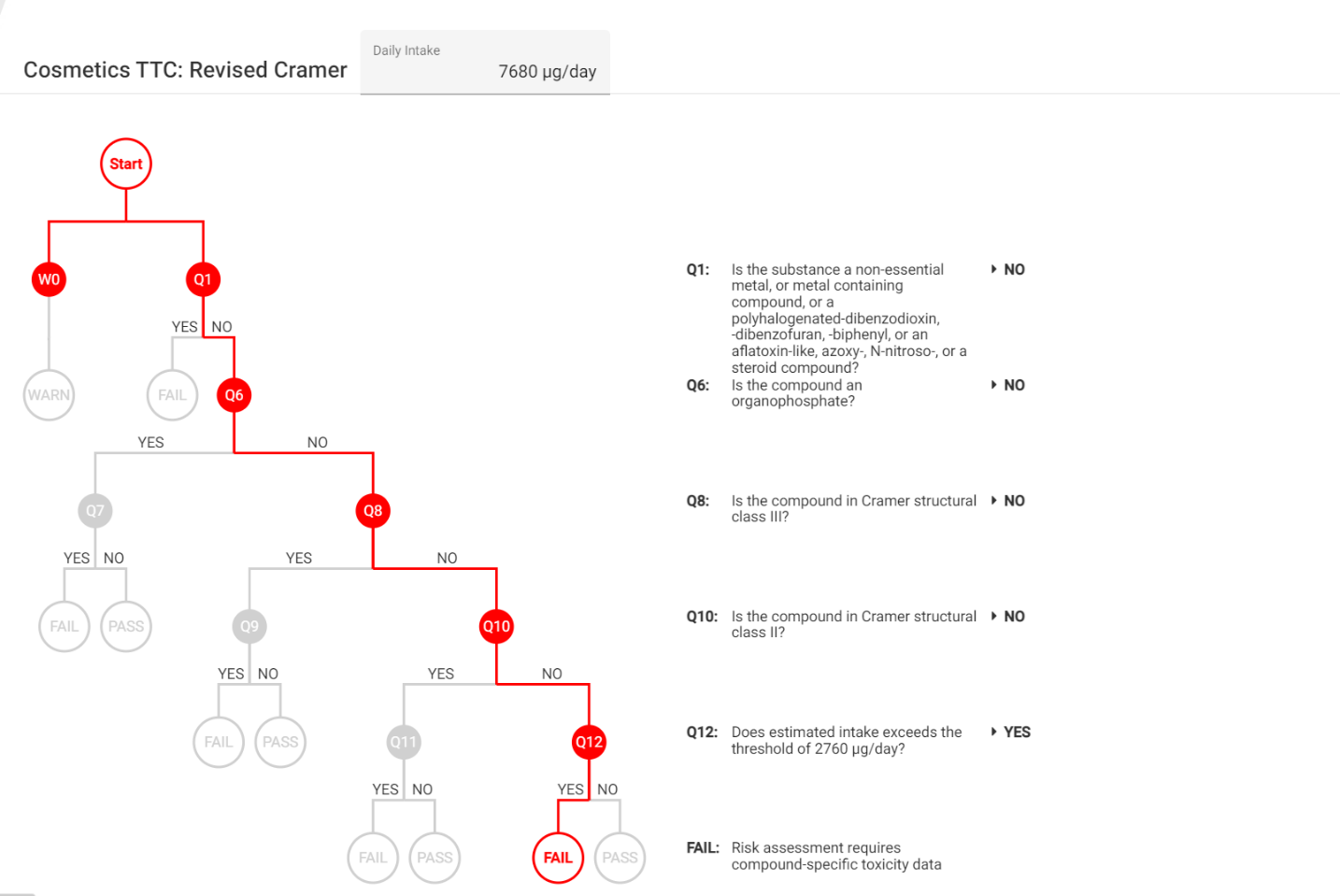


**S2.3 *In vitro* skin penetration**

Fresh viable pig skin (without hot steam treatment, by-product from meat industry) was obtained from a local, commercial butcher (Hammoor, Germany). The back skin was from female pigs (bred from “Dänische Landrasse” (female) and Pietrain (male) pigs, ~130 days old, 100 kg body weight). Pig skin samples were gently dry-shaved and washed with water before use. For the experiment, 2 replicate discs from each of 3 donors were used.

Skin of full-thickness was mounted on static Franz cells with a 4.9 cm² application area. The receptor compartment of the diffusion cell was filled with approx. 5 ml RF (1% BSA, 0.9% NaCl, 0.05% gentamycin sulfate in water), above a magnetic stirrer set to low rotation. The solubility of BSal was tested in the RF to confirm sink conditions. The experiment was conducted using non-occluded conditions. The temperature of the diffusion chamber and skin was maintained throughout the assay at 32 ± 2°C. The skin integrity was confirmed before application of BSal by measuring the impedance in PBS (all values were above the limit of 3 kOhm).

A dose of 3-4 mg/cm^2^ of the final formulation containing 0.5% BSal was applied to the surface of the skin and spread with the fingertip (without pressure) to ensure an even distribution of the cream. The RF was sampled after 24 h. The RF samples were transferred to a tube and the volume adjusted to 10 ml with acetonitrile (precipitation of proteins) and then filtered into HPLC-vials. After 24 h, the skin surface was washed with 4 ml methanol and cotton buds to remove residual test chemical. The solvent and cotton buds were transferred to a tube. A total of 16 tape strips were removed using tesaFilm, with the first tape strip measured separately, and tape strips 2-16 pooled. Tape strip 1 was transferred to 4 ml methanol and tape strips 2-16 were transferred to 40 ml solvent. The epidermis and dermis were separated by placing the skin disc with its epidermis side down on a heater covered with aluminium foil and heated at 70°C for 30 s. Afterwards, the epidermis was carefully removed using a spatula and tweezers. The epidermis was extracted in 4 ml methanol. The dermis was cut into small pieces and extracted in 10 ml methanol. All samples were sonicated for 10 min in a sonication bath, filtered and frozen prior to analysis.

The dermal absorption was calculated as the total amount present within epidermis (without the amount in the total SC strips), dermis and RF.

**S2.4 *In silico* prediction of metabolites**

*In silico* predictions of the metabolites were performed using Meteor Nexus version 3.1.0 (Lhasa Ltd.) using “Site of Metabolism Scoring (with Molecular Mass Variance)” with a relative threshold of 70 as reasoning methodology (default setting) and GLORYx. (de Bruyn Kops et al. 2021; Stork et al. 2020)

**S2.5 *In vitro* metabolism using PHH suspensions and cultures**

PHHs (5 donor pool, mixed gender, Batch TMW from BioIVT Westbury, NY, USA), at a density of 0.8 million cells/ml (0.25 ml final volume) in WME supplemented with 25 mM HEPES and 2 mM L-glutamine, were incubated with test compound (20 µM BSal, 20 µM SA or 5 µM BAlc) for 0, 30, 90, 120 and 240 min. Reference controls for low, medium and high clearance compounds were tolbutamide, midazolam and naloxone, respectively, all incubated at 5 µM. A reference chemical to demonstrate metabolic function, 5 µM 7-ethoxycoumarin, was incubated in parallel and samples were taken at 0 and 240 min. Additional negative controls (sampling at 0 and 240 min) were performed in the absence of PHH or heat-inactivated PHH to determine non-metabolic depletion of the parent compound. Samples were precipitated with an equal volume of acetonitrile containing internal standards (1 μM diazepam, 1 μM griseofulvin, 10 μM diclofenac) and then centrifuged (4800 x g, room temperature, 5 min) before analysis by UHPLC-MS/HRMS.

**S2.6 *In vitro* metabolism using liver S9 incubations**

Human hepatic S9 fractions from homogenized liver (pool of 200 donors, mixed gender, ref. 098H2610.S9) were purchased from Tebu-bio SAS (Le Perray-en-Yvelines, France). All incubations were performed in triplicate in microtubes in a 96-well tube format (1.2 mL, ABgene®, Thermo Fisher Scientific, Illkirch Cedex, France). Concentrations of 1 and 20 µM SA were incubated in a total volume of 1.2 mL liver S9 (2 mg/mL final concentration) supplemented with various cofactors (final concentrations: 1 mM NADPH, 2 mM UDP-glucuronic acid, 0.05 mM adenosine 3'-phosphate 5' phosphosulfate in sodium phosphate buffer (0.1 M, pH 7.4 with 2 mM MgCl_2_, 25 µg/ml alamethicin)). The incubation was carried out at 37°C in a shaking bath. At different time points (0, 30, 90, 120 and 240 min), 40 µL medium was removed and transferred to a well of a 96-well plate and quenched with 40 µL acetonitrile containing diclofenac (internal standard). The samples were mixed for 10 sec and then centrifuged at 2200 xg for 5 min. After centrifugation, the supernatant was analyzed by UHPLC-MS/HRMS.

**S2.7 LC-HRMS analysis of BSal and its metabolites**

The equipment for the LC-HRMS analysis included: Vanquish Quaternary Pump, Vanquish Column Compartment and a Vanquish Split sampler (Thermo Fisher Scientific, USA). Mass spectrometry was performed on Q-Exactive Plus mass spectrometers (Orbitrap technology with accurate mass) equipped with an APCI interface (Thermo Fisher Scientific, USA); data handling with the standard software Chromeleon version 7.2 SR5 muf. MS and chromatographic parameters (LC-MS/MS) are summarized in Supplementary Table S4. The pump flow rate was 600 μl/min and the analytes were separated on an YMC Triart C18 100A analytical column, 3.0 μm, 100x3.0 mm (RP283, YMC Europe GmbH) with a corresponding pre-column using the gradients for mobile phases as follows: 0.0 min = 30% A; 0.5 min = 30% A; 1.0 min = 60% A; 1.2 min = 97% A; 2.5 min = 97% A; 2.6 min = 30% A; 5.0 min = 30% A. Mobile Phase A was 100% acetonitrile and Mobile Phase B was H_2_O / 0.04% acetic acid. The reference item was separated on a Poroshell HPH-C18 analytical column, 2.7 μm, 100x3.0 mm (Agilent Technologies) with a corresponding pre-column using the gradients for mobile phases C (CAN / 0.1% (v:v) formic acid) and D (H_2_O / 0.1% (v:v) formic acid) as follows: 0.0 min = 95% D; 0.1 min = 95% D; 0.4 min = 3% D; 2.2 min = 3% D; 2.3 min = 95% D; 4.0 min = 95% D. As MS tune file, a generic tune file was used and as a lock mass for internal mass calibration, the [M+H]+ and [M-H]- ion of the diisooctyl phthalate (m/z 391.28429), which is ubiquitously present in the solvent system, was used. The quantification was performed in the full Scan mode with negative or positive ionization, the accurate mass of the monitoring ions ±5 mDa were used for test item and internal standard peak integration. Further analyzer settings were as follows: max. trap injection time 80 ms (Full Scan), sheath gas 45, aux gas 20, sweep gas 2, spray voltage 5 kV, capillary temperature 350°C, heater 350°C.

**Supplementary Table S4** MS and chromatographic parameters (LC-MS/MS)

| **Compound** | **Molecular weight (g/mol)** | **Quantitation ion [M+H]+ or *[M+H]-*(m/z)** | **Scan time (s)** | **Mass resolution** | **RT (min)** |
| --- | --- | --- | --- | --- | --- |
| Benzyl salicylate | 228.24 | *227.070* | 50.000 – 400.000 | 70,000 | 3.7 |
| Salicylic acid | 138.12 | *137.023* |  |  | 2.0 |
| Benzyl alcohol | 108.14 | 91.056 |  |  | 2.3 |
| Benzaldehyde | 106.12 | 107.050 |  |  | 2.9 |
| Benzoic acid | 122.12 | *121.028* |  |  | 2.6 |
| Hippuric acid | 179.17 | *178.05* |  |  | 1.2 |
| Diazepam | 284.7 | 285.079 |  |  | 3.3 |
| Griseofulvin | 352.8 | 353.079 |  |  | 3.1 |
| 7-Ethoxycoumarin | 190.19 | 191.0703 | 100 – 500 | 70,000 | 2.2 |
| 7-OH-Coumarin | 162.1 | 163.0390 |  |  | 1.9 |
| Diclofenac | 318.13 | 296.024 |  |  | 2.3 |

The amount of compound in the samples was expressed as percentage of remaining compound compared to time point zero (=100%). These percentages were plotted against the corresponding time points. *In vitro* intrinsic clearance (CLint) and half-life (t_1/2_) estimates were determined using the rate of precursor disappearance and following formula, based on the well-stirred liver model (Obach 1999):

**Equation 1**: t_1/2_ $=\frac{ln2}{-k}$

t_1/2_ = half-life [min]; k = slope from the linear regression of log [test compound] versus time plot [1/min]

**Equation 2**: CL int=(-k) * V * fu

Where CL_int_ = in vitro intrinsic clearance [μl/min/10^6^ cells]; t_1/2_ = half-life [min]; k = slope of the linear regression of log [test compound] versus time plot [1/min]; V = ratio of incubation volume and cell number and fu = unbound fraction in the blood. Since fu is unknown for the test compound, the calculation was performed with fu =1.

CL_int_ was used to calculate *in vivo* intrinsic clearance (CL_int_ *_in vivo_*) using Equation 3 and the scaling parameters, 25.7 g liver weight/body weight, 21 ml/min/kg liver blood flow and 120 million hepatocytes/g liver.

**Equation 3**: CL_int_ *_in vivo_* = CL_int_* W_liver_ *cd

CL_int in vivo_ = *in vivo* intrinsic clearance [ml/min/kg]; CL_int_ = *in vitro* intrinsic clearance [ml/min/million cells], W_liver_ = liver weight [g/kg]; cd = liver cell density [million hepatocytes/g liver]

Hepatic clearance (CLhep) was calculated as follows:

**Equation 4**: CL_hep_ = $\frac{CLint in vivo*Q}{CLint in vivo+Q}$

Where CL_hep_= hepatic clearance [ml/min/kg] and CL_int_ *_in vivo_* = *in vivo* intrinsic clearance [ml/min/kg]; Q = blood flow [ml/min/kg]

**S2.8 *In vitro* transporter studies for SA**

*Vesicular transport assays*

For vesicular transport assays​ inside out membrane vesicles derived from HEK293 cells expressing MDR1, MRP2 or MRP4 were incubated in transport buffer containing sucrose with SA or the probe substrate, in the presence or absence of a reference inhibitor (Supplementary Table S3A). Control vesicles were from mock-control HEK293 cells. The membrane vesicle suspension was mixed with ice-cold transport buffer, then the probe substrate solution was added (Reaction mixture, 50 µL per well).​ SA was added to the reaction mixture at 1.5-times the final assay concentration.​ Reaction mixtures and the start reagent (Mg-ATP or AMP in transport buffer) were pre-incubated separately for ​15 min to reach 37 ℃.​ The reaction was started by the addition of the start reagent (25 µL) to the reaction mixture (diluting it to the final assay concentration) and incubated for the specified incubation time (2 and 20 min with SA).​ After the incubation time, the reaction was quenched by the addition of 200 µL ice-cold washing mix to each well.​ The samples were then transferred to a filter plate and washed (5-times with 200 µL ice-cold washing mix) to remove any free SA or probe substrate.​ The amount of SA inside the filtered vesicles (or on the filter plate) was determined by LC-MS/MS.​

*HEK293 cell transporter assays​*

For uptake transporter assays​ HEK293 cells expressing OAT1, OAT2 and OAT3 were incubated in HBSS (pH 7.4) with SA or the probe substrate, in the presence or absence of a reference inhibitor (Supplementary Table S5B). Control cells were mock-control HEK293 cells. SA was diluted in the appropriate assay buffer at the final concentrations and pre-warmed separately on a helper plate at 37 ℃ for about 15 min.​ Prior to the experiments, cell culture medium was removed, the cells were rinsed and incubated for 15 min at 37 ℃ with assay buffer.​ Uptake experiments were carried out at 37 ℃, in assay buffer containing SA. Organic solvent concentrations were equal in all wells and did not exceed 1.5% (v/v).​ Following the incubation time (2 and 20 min with SA), cells were rinsed with assay buffer and lysed with methanol:assay buffer (2:1).​ The amount of substrate in the lysate was determined by LC-MS/MS.​

**Supplementary Table S5** Probe substrates, inhibitors, incubation time and acceptance criteria for (A) vesicular transport assays and (B) uptake transporter assays. NMQ: N-methyl quinidine; E217βG: Estradiol-17-β-glucuronide; DHEAS: Dehydroepiandrosterone​; HBSS: Hank’s Balanced Salt Solution, E3S: estrone-3-sulfate​.

1. Vesicular transport assays​

| **Transporter** | **Probe substrate**​ | **Reference Inhibitor**​ | **Incubation time**​ | **Fold acceptance**​**criteria**​ |
| --- | --- | --- | --- | --- |
| Human MDR1 | NMQ​  (1 µM)​ | Valspodar​  (1 µM)​ | 1 min​ | ≥ 5​ |
| Human MRP2 | E_2_17βG​  (100 µM)​ | Benzbromarone​  (200 µM)​ | 5 min​ | ≥ 10​ |
| Human MRP4 | DHEAS  (0.5 µM)​ | MK-571​  (150 µM)​ | 1.5 min​ | ≥ 5​ |

1. Uptake transporter assays

| **Transporter** | **Probe substrate**​ | **Reference Inhibitor**​ | **Incubation time**​ | **Fold**​  **Acceptance**​  **Criteria**​ |
| --- | --- | --- | --- | --- |
| Human OAT1 | Tenofovir​  (5 µM)​ | Probenecid​  (300 µM)​ | 2 min​ | ≥ 30​ |
| Human OAT2v1​ | cGMP​  (1.5 µM)​ | Indomethacin​  (300 µM) ​ | 1 min​ | ≥ 30​ |
| Human OAT3 | E3S​  (1 µM)​ | Probenecid​  (500 µM)​ | 1 min​ | ≥ 25​ |

*Fresh primary human proximal tubule cell assays*

Primary proximal tubule cells (PTCs) were isolated from kidneys from three human donors according to Brown et al. (2008). Once acceptable confluence and transepithelial electrical resistance (TEER) were reached, the monolayers were washed with modified Krebs buffer and incubated for ~30-min in the absence and presence of 200 µM Probenecid before the addition of test chemical. SA was tested at 1, 10 and 100 µM in the apical-to-basolateral (A-B) and basolateral-to-apical (B-A) directions. Each condition was assessed in three technical replicates in each of the three donors. SA was co-treated with Lucifer Yellow to account for paracellular leakage, so that the contribution of transcellular transport of the compound could be derived. Samples of the donor compartment were taken at the beginning and end of the incubation, and the receiver compartment was sampled at 30, 60 and 120 min. The intracellular content was assessed at the final timepoint by lysing the cells with 0.1% TFA (final concentration 0.01%). Samples were collected and stored at -80°C before being analyzed by mass spectrometry.

^14^C-P-aminohippurate (PAH) was tested at a single concentration of 10 μM in the absence and presence of the organic anion transporter (OAT) inhibitor, probenecid. This compound was sampled at the same timepoints as those for SA, to assess the functionality of the monolayers with respect to PAH transport. Samples were collected in triplicate and analyzed by liquid scintillation counting. For monolayers incubated with ^14^C-PAH, paracellular leakage was accounted for by co-incubating with ^3^H-Mannitol (paracellular marker *in vitro*), which could be counted within the same samples as the ^14^C-PAH.

**S2.9 Toxicity prediction of parent compound and metabolites**

The *in silico* toxicity tools in the software program, ToxPanel, was used to predict the toxicity of BSal, SA, BA, and hippuric acid. The endpoints investigated relevant for systemic toxicity were genotoxicity, endocrine disruption, reproductive toxicity, developmental toxicity and carcinogenicity and are listed in Supplementary Table S6.

**Supplementary Table S6** *In silico* tools used to predict the toxicity of BSal, SA, BA, and hippuric acid.

| **Endpoint** | ***In silico* tools** | **Version (7.10.2020)** |  |
| --- | --- | --- | --- |
|  |  |  |  |
| Endocrine Disruption | ADMET Predictor | 10.0.0.10 |  |
|  | ADMETsar | 2 |  |
|  | Case Ultra |  |  |
|  | Danish | Given by date of use |  |
|  | Derek nexus | 6.1.0 |  |
|  | Endocrine Disruptome | 2014 |  |
|  | NREDA | 1.1.1 |  |
|  | OPERA | 2.6.1 |  |
|  | QSAR toolbox | 4.4.1 |  |
|  | Vega | 1.1.5 |  |
| Developmental Toxicity | Case Ultra |  |  |
|  | Danish | Given by date of use |  |
|  | Derek nexus | 6.1.0 |  |
|  | QSAR toolbox | 4.4.1 |  |
|  | T.E.S.T. | 4.2.1 |  |
|  | Vega | 1.1.5 |  |
| Reproductive Toxicity | ADMET Predictor | 10.0.0.10 |  |
|  | Case Ultra |  |  |
|  | Danish | Given by date of use |  |
|  | Derek nexus | 6.1.0 |  |
|  | QSAR toolbox | 4.4.1 |  |

**S2.10 Cell stress assay**

Analysis of potential affected cell stress pathways was conducted according to protocols described previously (ter Braak et al. 2021; Wink et al. 2017; Wink et al. 2014; Wink et al. 2018). The cytotoxicity of 14 concentrations up to 10 mM of SA were measured as a fraction of Propidium Iodide (PI, a stain for late apoptosis/necrosis) positive cells after 24 h treatment. Based on these data, appropriate concentrations were selected for the ToxProfiler reporter assay.

The ToxProfiler assay uses human HepG2 reporter cell lines to study chemically induced activation of seven distinct stress pathways: oxidative stress (SRXN1-GFP), genetic stress (p21-GFP), ER stress (CHOP-GFP), autophagy (LC3-GFP), ion stress (MT1X-GFP), protein stress (HSPA1B-GFP) and inflammation (ICAM1-GFP). The seven independent reporter cells, in which biomarkers of the cell stress response pathways have been tagged with a GFP fluorophore, were cultured in DMEM medium supplemented with 10% FBS and 100 U/mL penicillin/streptomycin. A total of 9,000 cells per well was seeded in uncoated 384-well imaging plates. Two days after seeding, the DNA stain Hoechst was added. After 1 h incubation, the Hoechst medium was removed and the cells were treated with test compound in fresh cell culture medium containing the PI stain. Cells were imaged using a live cell confocal imager (Nikon Ti system) 24 h after treatment. Two images per well were taken (technical replicates) using three different channels (GFP; stress response activation, Hoechst: nuclei, PI: cell death). The GFP levels of the ToxProfiler reporters were quantified in their expected subcellular location and determined on a single cell level using automated segmentation pipelines. Positive reference treatments were included in the assay according to (ter Braak et al. 2021; Wink et al. 2017; Wink et al. 2014; Wink et al. 2018). For the analysis of the images, an image segmentation and analysis pipeline was applied that returns the GFP/PI induction in a specific sub-cellular localization and per individual cell. Point of departures (PoDs) were calculated using a custom Rpackage “modelpod”. Concentration response curves were fitted with loess regression. The intersect of this fitted curve with the mean of the solvent control plus two times the standard deviation of the concentration-response curve regression gives a value corresponding to the lowest concentration at which we observe a significant (positive or negative) effect (the PoD).

The ToxProfiler assay is considered to have a positive response when a PoD was calculated. The PoD were determined not only for reporter responses, but also for the PI (cytotoxicity) and increased cell count (mitogenicity) measurements. For each reporter a positive reference control was included, the respective GFP induction of this compound should have a value of at least 2x the standard deviation above the background GFP response induced by the solvent. For cytotoxicity we apply a threshold of 15% PI positive cells. These thresholds are based on historical control data.

**S2.11 Pharmacology profiling assays**

SA was tested at 10 µM in a panel of 83 assays for a range of pharmacological targets (listed in Supplementary Table S7). The list of targets included the original panel of 44 targets (including receptors, ion channels, transporters and enzymes) recommended by 4 major pharmaceutical companies as significant liabilities in drug development (Bowes et al. 2012), together with additional targets that have been reasoned to link to pathological effects. Results showing an inhibition or stimulation higher than 50% are considered to represent significant effects (i.e. a “hit”). Inhibition (or stimulation) between 25% and 50% are indicative of weak to moderate effects and results showing an inhibition (or stimulation) lower than 25% are not considered significant and mostly attributable to variability of the signal around the control level.

**Supplementary Table S7** Pharmacology profiling assay panel.

| **ASSAY NAME** |
| --- |
| 5-HT transporter (h) (antagonist radioligand) |
| 5-HT1A (h) (agonist radioligand) |
| 5-HT2A (h) (agonist radioligand) |
| 5-HT2B (h) (agonist radioligand) |
| 5-HT3 (h) (antagonist radioligand) |
| A1 (h) (antagonist radioligand) |
| A2A (h) (agonist radioligand) |
| ACE (h) |
| ACE-2 (h) |
| acetylcholinesterase (h) |
| alpha 1A (h) (antagonist radioligand) |
| alpha 2A (h) (antagonist radioligand) |
| alpha 2B (h) (antagonist radioligand) |
| AMPA (agonist radioligand) |
| AR(h) (agonist radioligand) |
| Aromatase / CYP19A1 |
| AT1 (h) (antagonist radioligand) |
| ATPase (Na+/K+) |
| beta 1 (h) (agonist radioligand) |
| beta 2 (h) (antagonist radioligand) |
| BZD (central)(h) (agonist radioligand) |
| Ca2+ channel (L, dihydropyridine site) (antagonist radioligand) |
| Ca2+ channel (L, diltiazem site) (benzothiazepines) (antagonist radioligand) |
| carbonic anhydrase II (h) |
| CB1 (h) (agonist radioligand) |
| CB2 (h) (agonist radioligand) |
| CCK1 (CCKA) (h) (agonist radioligand) |
| COX1(h) |
| COX2(h) |
| D1 (h) (antagonist radioligand) |
| D2S (h) (agonist radioligand) |
| dopamine transporter (h) (antagonist radioligand) |
| ERbeta Human Estrogen NHR Binding (Agonist Radioligand) Assay, Panlabs |
| Estrogen ER alpha (h) (agonist radioligand) |
| ETA (h) (agonist radioligand) |
| ETB (h) (agonist radioligand) |
| GR (h) (agonist radioligand) |
| H1 (h) (antagonist radioligand) |
| H2 (h) (antagonist radioligand) |
| HMG-CoA Reductase Human Enzymatic Assay, Panlabs |
| KATP channel (antagonist radioligand) |
| KV channel (antagonist radioligand) |
| M1 (h) (antagonist radioligand) |
| M2 (h) (antagonist radioligand) |
| MAO-A (antagonist radioligand) |
| MT2 (ML1B) (h) (agonist radioligand) |
| mu (MOP) (h) (agonist radioligand) |
| N neuronal alpha 4beta 2 (h) (agonist radioligand) |
| Na+ channel (site 2) (antagonist radioligand) |
| NMDA (antagonist radioligand) |
| norepinephrine transporter (h) (antagonist radioligand) |
| NTS1 (NT1) (h) (agonist radioligand) |
| PDE3A (h) |
| PDE4D2 (h) |
| Potassium Channel hERG (human)- [3H] Dofetilide |
| PPARalpha (h) (agonist radioligand) |
| PPARgamma h) (agonist radioligand) |
| PR (h) (agonist radioligand) |
| PXR (h) (agonist radioligand) |
| RARalpha (h) (agonist radioligand) |
| SKCa channel (antagonist radioligand) |
| Steroid 5 alpha-Reductase Rat Enzymatic Assay, Panlabs |
| TR β Human Thyroid Hormone NHR Binding (Agonist Radioligand) Assay, Panlabs |
| V1a (h) (agonist radioligand) |

**S2.12 Transcriptomics**

HepG2 (human hepatoblastoma) and MCF-7 (human Caucasian breast adenocarcinoma) cells were supplied by Public Health England European Collection of Cell Cultures (ECACC, Salisbury, UK). SA was prepared in DMSO as serial dilutions, resulting in concentrations in media of 10, 30, 100, 300 and 1000 µM with 0.1% DMSO used as a vehicle control. Trichostatin A served as positive control and was prepared in DMSO as serial dilutions, resulting in concentrations in media of 0.01, 0.03, 0.1, 0.3 and 1 µM with 0.1% DMSO used as a vehicle control. Three biological replicates were treated with the appropriate dose of compound for 6 h and 24 h, respectively, prior to cell lysis. Six biological replicates were used for the vehicle control. Samples were analyzed with Illumina RNA-sequencing technology.

***Analysis method 1***

*Differential Gene Expression (DGE)*

Input data consists of tables of count data for reads mapped to the Hs38 Ensembl human reference genome. Counted reads for each annotated CDS feature were analyzed for differential expression in R (v.3.5.3) using the BioConductor library DESeq2 (v.1.20.0) (Love et al. 2014). The final output of DESeq2 is a table of estimated Log2 fold change, p-values for the defined contrasts tested, as well as Benjamini-Hochberg corrected false discovery p-values (FDR) (Benjamini and Hochberg 1995). The significance of differential expression was determined using several criteria, either singly or in combination. A statistical criterion was used, the Benjamini and Hochberg step-up false discovery rate corrected p-value of less than 0.05 (FDR<0.05), a commonly used threshold in whole transcriptomic analysis. A magnitude of change threshold of 1.5-fold was used, this degree of change is well within the detection range of RNA-Seq experimentation and was applied as the absolute value of estimated fold change (|FC|>1.5). Finally, the application of both a statistical threshold and a magnitude of change threshold (FDR<0.05 & |FC|>1.5 fold) was used.

*Benchmark dose analyses*

Gene expression data from RNA-Seq experiments were analyzed using the BMDExpress software version 2.2 (BMDExpress2 is publicly available through an NIEHS repository (github.com/auerbachs/BMDExpress-2)). First, normalized Log2-transformed expression values were computed for each gene (or annotated feature) in an RNA-Seq experiment. These data were then fit to a series of dose-response models: linear model, Hill model, Power model, second-degree polynomial model and second degree exponential model. Each gene was fit independently using the BMDExpress software program which in turn utilized models developed by the EPA’s BMDS group. Each model is computed assuming constant variance as the data is log2 transformed before analyses which results in a data distribution that has at least approximately constant variance. The benchmark response (BMR) factor was set to 1 SD computed using the entire fitted curve across all doses. To avoid model extrapolation, genes with a BMD value greater than the highest concentration used in the experiment were removed from further analysis, as were poor-fitting best overall models with a goodness of fit p-value less than or equal to 0.1. A BMDU/BMDL ratio of greater than 40 was also grounds for the rejection of a best-fitting model, as this indicates an increasingly large 95% CI about the estimate. The result is a single best-fitting dose-response model for each gene, and a measure of both the BMD_1SD_ and its upper (BMDU) and lower (BMDL) 95% confidence limits.

The final selection of genes with the best models passing these quality control thresholds were then matched to elements in an ontology (i.e., the GO and Reactome). In this study, any ontology category with at least 5 or more best model elements found amongst the defined ontology category elements and with a Fisher’s exact test p-value < 0.05 was considered enriched. The genes associated with each enriched pathway were then used to estimate pathway or category BMD_1SD_ and BMDL by averaging (or using the median value of) the estimates for the individual elements found.

***Analysis method 2***

*Alignment*

FastQC2 was run on all RNA-seq samples to ensure adequate per base quality and per base N content. The RNA-seq reads were aligned to the hg38 genome using the STAR aligner (Dobin et al. 2012). After alignment, the total sequenced reads, alignment rate, number of aligned reads and percentage of uniquely aligned reads were computed for each sample.

*Normalization*

Expression values for each probe were normalized by applying DESEQ2 normalization. A pseudo-read-count of 1.0 was added to each normalized expression value, and then the values were log2 transformed.

*Batch Correction for MCF7 6 h data*

Principal Component analysis on the expression data was performed to determine if there was a batch effect. The PCA indicated a clear separation of data by library batch. Library batches C and D separated from E and F on principal component plots. On further investigation of the experimental settings, it was observed that the batches E and F had 2 or more times the RNA concentration compared to batches C and D. Therefore, batch correction was applied to the data prior to performing downstream analysis.

*Outlier Detection*

For each study group, principal component analysis, hierarchical cluster analysis and inter-replicate correlation analysis was performed. This was used along with the per sample QC metrics to determine outlier samples.

*Gene Analysis*

We utilized Student’s t-test statistics to measure gene-level differential activity. The significance p-values, false detection rate, and familywise error rate (FWER) for gene level activity were computed using 10,000 random permutations of sample labels. False detection rate and FWER calculations were performed separately for all signatures within each subcategory. For a gene to be considered as a significantly differentially expressed gene (i.e. DEG), it was required to have an absolute foldchange ≥ 1.5 and Global Permutation based P-value ≤ 0.005.

*Dose Response Analysis*

Dose response analysis was performed using BMDExpress version 3.04 (Phillips et al. 2018). The following models were used for dose response curve fitting: Power, Linear (Poly1), Polynomial 2, Exponential 2, Exponential 3, Exponential 5, Hill. Dose response analysis was performed with the benchmark response (BMR) factor of 1 SD. The following criteria was used to determine probes with a strong monotonic increasing/decreasing trend: William’s trend test adjusted p-value ≤ 0.05, Max fold change ≥ 1.5, BMDU/BMDL ≤ 40, BMD ≤ highest dose. Those genes were then used in categorical (pathway) analysis for the Gene Ontology (GO) terms and REACTOME pathways. To determine significantly dose responsive pathways, the following criteria was used: Fisher’s exact right tail p-value ≤ 0.05, Pathway coverage ≥ 10% and number of significant dose responsive genes in pathway ≥ 3.

**S2.13 ReproTracker**

SA was tested in the ReproTracker according to Jamalpoor et al. (2022). Test concentrations were identified in a pilot study in which 20 concentrations, increasing by 2-fold, up to 1 mM were tested in human induced pluripotent stem cells (iPSCs). Cytotoxicity was assessed using the AlamarBlue cell viability assay performed after 7 days. Positive and negative assay controls were 5-fluorouracil (cytotoxic) and saccharin (non-cytotoxic and non-teratogenic).

For the main study, iPSCs were differentiated into cardiomyocytes, hepatocytes and neural rosettes, during which time, they were exposed to 5 concentrations of SA (in 0.1% v/v final concentration of DMSO) for 14 days (cardiomyocytes), 21 days (hepatocytes) or 13 days (neural rosettes) of differentiation. Morphology was closely monitored on daily basis for identifying any possible disruption of proper stem cell differentiation following SA exposure. RNA samples were collected on Day 0 (D0), D7 and D14 or D21 for biomarker expression analysis using multiplex qRT-PCR. Biomarkers were OCT4, BMP4 and MYH6 (cardiomyocytes); OCT4, FOXA2 and AFP (hepatocytes) and OCT4, SOX1 and PAX6 (neural rosettes). Alterations in expression pattern of the tissue-specific biomarkers were compared to solvent control cultures.

Thalidomide and saccharin were included as positive and negative controls for heart and liver differentiation assays, respectively. Retinoic acid and saccharin were included as positive and negative controls for neural rosette differentiation assays, respectively.

A chemical is defined as a teratogen when it reduces the expression of the biomarker genes below the set threshold level in a concentration-dependent manner, upon exposure to a minimum of 2 consecutive non-cytotoxic concentrations (indicating disruption of the developmental). In addition, morphological abnormalities and a decline in contraction of cardiomyocytes in response to chemical exposure is an indication of teratogenicity.

**S2.14 Nephrotoxicity in primary human proximal tubule cells**

Primary proximal tubule cells (PTCs) from 4 donors were isolated from kidneys from three human donors according to Brown et al. (2008). Once acceptable confluence and transepithelial electrical resistance (TEER) were reached in 96-well Transwell plates, cells were treated with a range of concentrations of SA for 72 h at 37°C and 5% CO_2_. After this times, TEER, cellular ATP content and LDH leakage into the medium from the apical supernatant and cells were measured. Supernatants were collected for biomarker (KIM-1, NGAL and clusterin) quantification according to Brown et al. (2008).

**S2.15 References**

Benjamini Y, Hochberg Y (1995) Controlling the False Discovery Rate: A Practical and Powerful Approach to Multiple Testing. Journal of the Royal Statistical Society: Series B (Methodological) 57(1):289-300 doi:<https://doi.org/10.1111/j.2517-6161.1995.tb02031.x>

Bowes J, Brown AJ, Hamon J, et al. (2012) Reducing safety-related drug attrition: the use of in vitro pharmacological profiling. Nat Rev Drug Discov 11(12):909-22 doi:10.1038/nrd3845

Brown CD, Sayer R, Windass AS, et al. (2008) Characterisation of human tubular cell monolayers as a model of proximal tubular xenobiotic handling. Toxicol Appl Pharmacol 233(3):428-38 doi:10.1016/j.taap.2008.09.018

de Bruyn Kops C, Šícho M, Mazzolari A, Kirchmair J (2021) GLORYx: Prediction of the Metabolites Resulting from Phase 1 and Phase 2 Biotransformations of Xenobiotics. Chemical Research in Toxicology 34(2):286-299 doi:10.1021/acs.chemrestox.0c00224

Dobin A, Davis CA, Schlesinger F, et al. (2012) STAR: ultrafast universal RNA-seq aligner. Bioinformatics 29(1):15-21 doi:10.1093/bioinformatics/bts635

Jamalpoor A, Hartvelt S, Dimopoulou M, et al. (2022) A novel human stem cell-based biomarker assay for in vitro assessment of developmental toxicity. Birth Defects Res doi:10.1002/bdr2.2001

Love MI, Huber W, Anders S (2014) Moderated estimation of fold change and dispersion for RNA-seq data with DESeq2.” Genome Biology, 15, 550. doi:10.1186/s13059-014-0550-8.

Obach RS (1999) Prediction of human clearance of twenty-nine drugs from hepatic microsomal intrinsic clearance data: An examination of in vitro half-life approach and nonspecific binding to microsomes. Drug Metab Dispos 27(11):1350-9

Phillips JR, Svoboda DL, Tandon A, et al. (2018) BMDExpress 2: enhanced transcriptomic dose-response analysis workflow. Bioinformatics 35(10):1780-1782 doi:10.1093/bioinformatics/bty878

Stork C, Embruch G, Šícho M, et al. (2020) NERDD: a web portal providing access to in silico tools for drug discovery. Bioinformatics 36(4):1291-1292 doi:10.1093/bioinformatics/btz695

ter Braak B, Wolters L, Osterlund T, Van de Water B, Hendriks G (2021) Validation of the ToxProfiler reporter assay for toxicological profiling and determination of the underlying mode of action. Toxicology Letters 350:S63-S64 doi:<https://doi.org/10.1016/S0378-4274(21)00397-0>

Wink S, Hiemstra S, Herpers B, van de Water B (2017) High-content imaging-based BAC-GFP toxicity pathway reporters to assess chemical adversity liabilities. Arch Toxicol 91(3):1367-1383 doi:10.1007/s00204-016-1781-0

Wink S, Hiemstra S, Huppelschoten S, et al. (2014) Quantitative high content imaging of cellular adaptive stress response pathways in toxicity for chemical safety assessment. Chem Res Toxicol 27(3):338-55 doi:10.1021/tx4004038

Wink S, Hiemstra SW, Huppelschoten S, Klip JE, van de Water B (2018) Dynamic imaging of adaptive stress response pathway activation for prediction of drug induced liver injury. Arch Toxicol 92(5):1797-1814 doi:10.1007/s00204-018-2178-z

Yang C, Rathman JF, Bienfait B, et al. (2023) The role of a molecular informatics platform to support next generation risk assessment. Computational Toxicology 26:100272 doi:<https://doi.org/10.1016/j.comtox.2023.100272>
